# The Combined Strategy for iron uptake is not exclusive to domesticated rice (*Oryza sativa*)

**DOI:** 10.1038/s41598-019-52502-0

**Published:** 2019-11-06

**Authors:** Andriele Wairich, Ben Hur Neves de Oliveira, Ezequiel Barth Arend, Guilherme Leitão Duarte, Lucas Roani Ponte, Raul Antonio Sperotto, Felipe Klein Ricachenevsky, Janette Palma Fett

**Affiliations:** 1 Programa de Pós-Graduação em Biologia Celular e Molecular, Centro de Biotecnologia, Universidade Federal do Rio Grande do Sul, Porto Alegre, Brazil; 2 Faculdade de Agronomia, Universidade Federal do Rio Grande do Sul, Porto Alegre, Brazil; 3 Departamento de Biologia, Centro de Ciências Naturais e Exatas, Universidade Federal de Santa Maria, Santa Maria, Brazil; 4 Programa de Pós-Graduação em Biotecnologia, Universidade do Vale do Taquari - Univates, Lajeado, Brazil

**Keywords:** Plant domestication, Plant stress responses, Plant molecular biology

## Abstract

Iron (Fe) is an essential micronutrient that is frequently inaccessible to plants. Rice (*Oryza sativa* L.) plants employ the Combined Strategy for Fe uptake, which is composed by all features of Strategy II, common to all Poaceae species, and some features of Strategy I, common to non-Poaceae species. To understand the evolution of Fe uptake mechanisms, we analyzed the root transcriptomic response to Fe deficiency in *O. sativa* and its wild progenitor *O. rufipogon*. We identified 622 and 2,017 differentially expressed genes in *O. sativa* and *O. rufipogon*, respectively. Among the genes up-regulated in both species, we found Fe transporters associated with Strategy I, such as *IRT1, IRT2* and *NRAMP1*; and genes associated with Strategy II, such as *YSL15* and *IRO2*. In order to evaluate the conservation of these Strategies among other Poaceae, we identified the orthologs of these genes in nine species from the *Oryza* genus, maize and sorghum, and evaluated their expression profile in response to low Fe condition. Our results indicate that the Combined Strategy is not specific to *O. sativa* as previously proposed, but also present in species of the *Oryza* genus closely related to domesticated rice, and originated around the same time the AA genome lineage within *Oryza* diversified. Therefore, adaptation to Fe^2+^ acquisition via IRT1 in flooded soils precedes *O. sativa* domestication.

## Introduction

Iron (Fe) is an essential micronutrient for virtually all organisms. In humans, anemia prevalence was one third of world population in 2010, and Fe deficiency anemia (IDA) is correlated with decreased cognitive performance, low weight at birth, and child and maternal mortality^1^. In plants, the capacity to change redox states from Fe^2+^ (ferrous) to Fe^3+^ (ferric) allows Fe to participate in electron transfer reactions in both photosynthesis, respiration and chlorophyll biosynthesis^2,3^. Higher plants cultivated under Fe deficiency experience severe chlorosis, reduction on biomass, yield and nutritional value of their grains^4^. In calcareous soils, which cover approximately one third of the Earth’s surface^4,5^, Fe is less bioavailable to plants, which can lead to Fe deficiency. In contrast, Fe can become harmful when present at elevated concentrations in plant tissues, since it can react with oxygen and catalyze the formation of reactive oxygen species through the Fenton reaction^6^. Thus, plants must tightly regulate internal Fe concentration to avoid both Fe toxicity and Fe deficiency^2^.

To cope with Fe deficiency, plants possess two different ways to maintain adequate levels of Fe^7^. Strategy I, or reduction strategy, found in all plants except those from the *Poaceae* family, consists in: (1) lowering soil pH by extrusion of H^+^ to increase Fe^3+^ solubility, which relies on P-type ATPases such as AtAHA2 in Arabidopsis^8^; (2) reduction of Fe^3+^ to Fe^2+^ at the root surface by a plasma membrane-bound ferric-chelate reductase, named AtFRO2 (Ferric Reductase Oxidase)^9^; and uptake of Fe^2+^ into root cells by the Fe high-affinity transporter AtIRT1 (Iron-Regulated Transporter)^10^. All proteins involved in this strategy increase their expression level under Fe deficiency. On the other hand, plants from the *Poaceae* family rely on Strategy II, or chelation strategy, to absorb Fe. This strategy employs the release of phytosiderophores into the rhizosphere. Phytosiderophores are derivatives of mugineic acid (MAs) family of modified amino acids^11^. MAs synthesis involves the trimerization of S-adenosyl Met to form nicotianamine (NA), catalyzed by nicotianamine synthase (NAS)^12^; the conversion of NA in 3-keto intermediate by the transfer of an amino group by nicotianamine aminotransferase (NAAT)^13^, and DMA synthesis by deoxymugineic acid synthase (DMAS)^13^. The first MA synthesized in the pathway is DMA, but different grass species may secrete other forms of MAs. Phytosiderophore secretion is performed by OsZIFL4/TOM1 (transporter of mugineic acid family phytosiderophores 1) in rice^14,15^. In the rhizosphere, phytosiderophores chelate Fe^3+^ and the formed complex Fe(III)-MA is transported into root cells through specific transmembrane proteins of the Yellow Stripe (YS) family, such as Yellow Stripe 1 (YS1) in maize^16^ and its ortholog Yellow Stripe-like 15 (OsYSL15) in rice^17^.

In spite of being a grass and relying on Strategy II, *O. sativa* was shown to induce the Fe^2+^ transporters *OsIRT1* and *OsIRT2* under Fe deficiency^18,19^. It was proposed that *O. sativa* uses a Combined Strategy (CS) to absorb Fe from the rhizosphere, using all features of Strategy II plants and partial features of Strategy I, such as IRT-type transporters^20^. The capability to absorb Fe^2+^ would have evolved in *O. sativa* as an adaptation to flooded paddies, since cultivated rice is well adapted for growth under submerged conditions, in which Fe^2+^ is frequently more abundant than Fe^3+^, unlike most graminaceous crops^11,18,21^. Thus, *O. sativa* is the only plant described as using the CS for Fe uptake to date^22^.

The *Oryza* genus is composed by two domesticated species, *O. sativa* and *O. glaberrima*, and 25 wild species which diverged from their wild progenitors 9,000 to 8,000 years ago^23^. The *Oryza* genus comprehends 11 genome types, 6 diploid (*n* = 12: AA, BB, CC, EE, FF, and GG) and 5 polyploid (*n* = 24: BBCC, CCDD, HHJJ, HHKK, and KKLL)^24^, and a genome variation size of 3.6 times^25^. *O. rufipogon*, an Asian wild grass, is the species most closely related to *O. sativa*^23^.

In the present study, a transcriptomic analysis was performed to compare the regulation of genes involved in Fe deficiency response in *O. sativa* and its wild progenitor *O. rufipogon*. We also analyzed the expression of the orthologs of *OsYSL15*, *OsIRT1*, *OsNRAMP1* and *OsIRO2* in other wild *Oryza* species, as well as in sorghum (*Sorghum bicolor*) and maize (*Zea mays*). Our results indicate that the CS observed in rice, based on OsYL15 and OsIRT1 as Fe^3+^ -phytosiderophore and Fe^2+^ transporters, is not an evolutionary novelty restricted to *O. sativa*, but has an origin that precedes the split of most AA genome *Oryza* species. This suggests a common origin for the CS in these species, and indicates that adaptation to Fe^2+^ acquisition in flooded soils precedes rice domestication.

## Results

### Plants of *O. sativa* and *O. rufipogon* show similar Fe deficiency physiological responses

**Figure 1:**
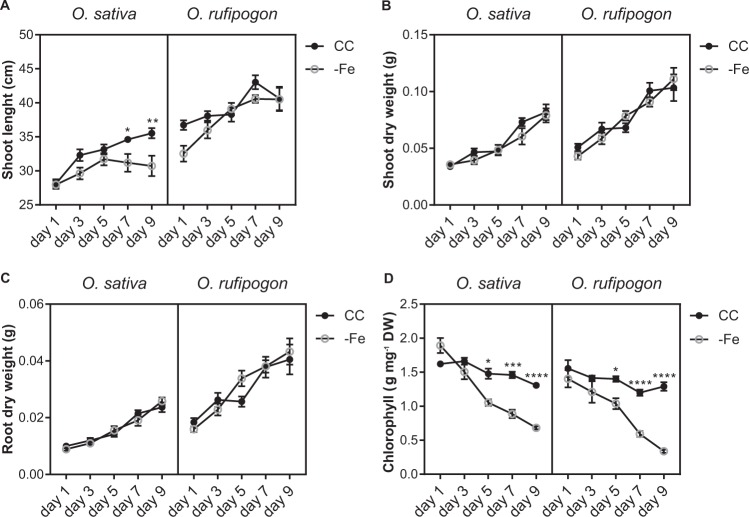
Phenotypic analysis of *Oryza sativa* and *Oryza rufipogon* plants grown under control (CC) and Fe deficiency (−Fe) conditions. Four week-old plants were grown with or without Fe for up to 9 days. (**A**) Shoot length (cm) (n = 10). (**B**) Shoot dry weight (g) (n = 10). (**C**) Root dry weight (g) (n = 10). (**D**) Chlorophyll concentration (µg mg^−1^ DW) (n = 4, 3 plants each). The *x*-axis represents days after onset of −Fe treatment. Values are the averages ± SE. Asterisks indicate statistical difference between plants grown under CC and −Fe conditions (Student *t*-test, *P-value < 0.05, **P-value < 0.01, ***P-value < 0.001, ****P-value < 0.0001). DW = dry weight.

Plants of *O. sativa* and *O. rufipogon* were cultivated under control condition (CC, containing 100 μM Fe^+3^-EDTA) and Fe deficiency (−Fe) in non-aerated hydroponics for nine days. Overall, both species responded similarly to low Fe conditions. Shoot length decreased significantly in *O. sativa* plants compared with plants in CC after seven days of −Fe, while no change was observed in *O. rufipogon* plants (Fig. 1A). Shoot and root dry weight showed no significant differences in both species when comparing plants under CC or −Fe conditions (Fig. 1B,C). Chlorophyll concentration decreased clearly in both species after five days of −Fe treatment (Fig. 1D). These data indicate that both *O. sativa* and *O. rufipogon* are at the early stage of the Fe deficiency response at five days of treatment.

### *O. sativa* and *O. rufipogon* transcriptomic changes under Fe deficiency

In order to compare the Fe deficiency gene expression responses in *O. sativa* and *O. rufipogon*, transcriptomic analyses of roots from plants submitted to CC and −Fe treatments were performed. Deep sequencing generated 33,253,905 reads from *O. sativa* CC libraries, 36,581,515 from *O. sativa* −Fe libraries; 34,973,923 from *O. rufipogon* CC libraries, and 36,365,135 from *O. rufipogon* −Fe libraries. Transcripts were considered differentially expressed when FDR < 0.05 (Supplementary Fig. 1). Comparing −Fe to CC, *O. sativa* showed a total of 622 differentially expressed genes (340 up- and 282 down-regulated), which represent 1.77% of all annotated genes in the genome of this species (Supplementary Table 1). *O. rufipogon* showed a total of 2,017 differentially expressed genes (1,433 up- and 584 down-regulated), which represent 5.43% of all annotated genes in the genome of this species (Supplementary Table 2).

### Functional annotation of differentially expressed transcripts associated with Fe deficiency response in *O. sativa* and *O. rufipogon* shows similar processes

Aiming to compare the transcriptomic changes in roots under −Fe from *O. sativa* and *O. rufipogon*, we performed Gene Ontology (GO) categories enrichment analysis. From a total of 622 and 2,017 differentially expressed genes in *O. sativa* and *O. rufipogon*, respectively, 485 and 1,516 genes could be assigned a GO term and were considered in the enrichment analysis. We found 216 terms enriched in the set of differentially expressed genes of *O. sativa*, with 120 terms up- and 96 terms down-regulated by −Fe, and 280 terms enriched in the set of differentially expressed genes of *O. rufipogon*, with 138 terms up- and 142 terms down-regulated. When comparing *O. sativa* and *O. rufipogon*, we found processes regulated in both: 26 processes were up- and 29 were down-regulated in the two *Oryza* species. Among the up-regulated processes in both species (Supplementary Fig. 2A), we found “iron ion transport”, “zinc II ion transport”, “organic acid biosynthetic process”, processes related to “amino acid salvage” including “L-methionine salvage” and “L-methionine salvage from methylthioadenosine” as well as several amino acid biosynthetic processes, mainly related to “methionine biosynthetic process”, “S-adenosylmethionine biosynthetic process” and “L-methionine biosynthetic process”. Among the commonly down-regulated processes (Supplementary Fig. 2B), we found terms associated to “cellular response to nutrient levels”, “cellular responses to starvation”, “responses to extracellular stimulus”. Four similar categories, including metabolic and biosynthetic processes related to glycolipid, glycosilceramide, glucosylceramide and glycosphingolipid; and three categories and processes related to sulfur transport, such as “sulfate transport”, “sulfur compound transport” and “sulfate transmembrane transport” were also identified.

GO terms exclusively up-regulated in *O. sativa* include “formate metabolic process”, “ureide catabolic process”, “ureide metabolic process”, “divalent metal ion transport” and “divalent inorganic cation transport”, whereas down-regulated terms exclusively in *O. sativa*, which include “chemical homeostasis”, “response to toxic substance”, “cellular oxidant detoxification” and “response to oxidative stress”, showed high level of significance (Supplementary Fig. 3A). *O. rufipogon* up-regulated GO terms include “nitrogen cycle metabolic process”, “nitrate assimilation”, “ammonium transport”, and “response to oxidative stress”, and down-regulated terms include “cellular nitrogen compound catabolic process”, “divalent metal ion transport” and “cellular response to phosphate starvation” (Supplementary Fig. 3B).

### The Fe deficiency regulon genes are responsive in both *O. sativa* and *O. rufipogon*

Although the number of differentially expressed genes was different in roots of *O. sativa* and *O. rufipogon* under −Fe, we found orthologous genes regulated in both species. Fifty-seven genes up-regulated by −Fe are orthologous between the two species, whereas 45 orthologous genes were down-regulated in both species after −Fe treatment (Table 1). Interestingly, a number of genes encoding proteins involved in Strategy I for Fe^2+^ uptake from the rhizosphere were up-regulated under −Fe in both species, such as *IRT1*, *IRT2* and *NRAMP1*. We also found genes related to Strategy II for Fe uptake which were up-regulated in both species, including enzymes involved in biosynthesis and secretion of mugineic acids (MAs), such as *S-adenosylmethionine synthase 2* (*SAM2*), *deoxymugineic acid synthase* (*DMAS*) and *nicotianamine synthase 1* (*NAS1*), and genes involved in the biosynthesis of methionine, as *MTK1* (*Methylthioribose kinase 1*), a precursor for the MAs biosynthetic pathway^13^. Known regulators of Strategy II, such as the iron-regulated bHLH transcription factor *IRO2*, which plays a role in transcriptional regulation of genes that participate in Fe acquisition^26^; *IRO3*, which plays an important role as a negative regulator of the Fe deficiency response in *O. sativa*; and *HRZ1* and *HRZ2*, which are iron-binding sensors that negatively regulate iron acquisition under conditions of Fe sufficiency^27^, were also up-regulated in roots of both species. Finally, the genes encoding the main Fe(III)-deoxymugineic acid transporter in roots (*YSL15*, which is essential for Fe uptake^17^), were also up-regulated in both *O. sativa* and *O. rufipogon*. These results strongly indicate that these two species utilize a common mechanism, the Combined Strategy, for Fe uptake.

**Table 1:** List of differentially expressed genes identified by RNAseq in response to Fe-deficiency in roots of *Oryza sativa* and *Oryza rufipogon*.

*O. sativa*	*O. rufipogon*	Gene name	Regulation	Description
**Phytosiderophore biosynthesis**				
OS01G0323600/LOC_Os01g22010	ORUFI01G14960	*SAM2*	up	S-adenosylmethionine synthase [Source:UniProtKB/TrEMBL;Acc:A0A0R7VIP9]
OS02G0306401/LOC_Os02g20360	ORUFI02G13810	*NAAT1*	up	Os02g0306401 protein [Source:UniProtKB/TrEMBL;Acc:A0A0P0VI36]
OS03G0237100/LOC_Os03g13390	ORUFI03G09980	*DMAS*	up	NADH-dependent oxidoreductase 1, putative, expressed [Source:UniProtKB/TrEMBL;Acc:Q10PE7]
OS03G0307300/LOC_Os03g19427	ORUFI03G15260	*NAS1*	up	Nicotianamine synthase 1 [Source:UniProtKB/TrEMBL;Acc:H9BE58]
OS04G0669800/LOC_Os04g57400	ORUFI04G30700	*MTK1*	up	Methylthioribose kinase 1 [Source:UniProtKB/Swiss-Prot;Acc:Q7XR61]
OS06G0112200/LOC_Os06g02220	ORUFI06G00790		up	Methylthioadenosine/S-adenosyl homocysteine nucleosidase [Source:UniProtKB/TrEMBL;Acc:Q9LHZ0]
**Iron homeostasis**				
OS01G0689451/LOC_Os01g49470	ORUFI01G30140	*HRZ1*	up	Hemerythrin motif-containing really interesting new gene (RING)-and zinc-finger protein 1 [Source:UniProtKB/TrEMBL;Acc:V9G2Z0]
OS05G0551000/LOC_Os05g47780	ORUFI05G26690	*HRZ2*	up	Os05g0551000 protein [Source:UniProtKB/TrEMBL;Acc:A0A0P0WPZ3]
OS03G0751100/LOC_Os03g54000	ORUFI03G35800	*OPT7*	up	Oligopeptide transporter 3, putative, expressed [Source:UniProtKB/TrEMBL;Acc:Q75LM0]
OS11G0151500/LOC_Os11g05390	ORUFI11G03210	*ENA1*	up	Os11g0151500 protein [Source:UniProtKB/TrEMBL;Acc:Q0IUK3]
OS12G0568500/LOC_Os12g38051	ORUFI12G18020		up	Metallothionein-like protein 1, putative, expressed [Source:UniProtKB/TrEMBL;Acc:Q2QNE5]
OS12G0570700/LOC_Os12g38270	ORUFI12G18180	*MT4A*	up	Metallothionein-like protein [Source:UniProtKB/TrEMBL;Acc:A1L4T7]
**Iron transport**				
OS02G0650300/LOC_Os02g43410	ORUFI02G27480	*YSL15*	up	Iron-phytosiderophore transporter YSL15 [Source:UniProtKB/Swiss-Prot;Acc:Q6H3Z3]
OS03G0667300/LOC_Os03g46454	ORUFI03G29890	*IRT2*	up	Fe(2+) transport protein 2 [Source:UniProtKB/Swiss-Prot;Acc:Q6L8G1]
OS03G0667500/LOC_Os03g46470	ORUFI03G29910	*IRT1*	up	Fe(2+) transport protein 1 [Source:UniProtKB/Swiss-Prot;Acc:Q75HB1]
OS04G0542200/LOC_Os04g45860	ORUFI04G21940	*YSL9*	up	Os04g0542200 protein [Source:UniProtKB/TrEMBL;Acc:A0A0P0WDA4]
OS07G0258400/LOC_Os07g15460	ORUFI07G09420	*NRAMP1*	up	Metal transporter Nramp1 [Source:UniProtKB/Swiss-Prot;Acc:Q0D7E4]
OS09G0396900/LOC_Os09g23300	ORUFI09G10010	*VIT2*	down	Os09g0396900 protein [Source:UniProtKB/TrEMBL;Acc:A0A0P0XMS4]
**Transcription factors**				
OS01G0952800/LOC_Os01g72370	ORUFI01G47560	*IRO2*	up	Os01g0952800 protein [Source:UniProtKB/TrEMBL;Acc:Q0JFZ0]
OS03G0379300/LOC_Os03g26210	ORUFI03G20000	*IRO3*	up	Helix-loop-helix DNA-binding domain containing protein, expressed [Source:UniProtKB/TrEMBL;Acc:Q10KL8]
OS07G0573300/LOC_Os07g38580	ORUFI07G20280	*zinc finger family protein*	down	Os07g0573300 protein [Source:UniProtKB/TrEMBL;Acc:Q6ZL20]
**Other transporters**				
OS08G0207500/LOC_Os08g10630	ORUFI08G06860	*ZIP4*	up	Zinc transporter 4 [Source:UniProtKB/Swiss-Prot;Acc:Q6ZJ91]
OS08G0369000/LOC_Os08g28170	ORUFI08G14240	*nucleobase-ascorbate transporter*	up	Os08g0369000 protein [Source:UniProtKB/TrEMBL;Acc:Q0J648]
OS09G0440700/LOC_Os09g26900	ORUFI09G12590	*COPT5.1*	up	Copper transporter 5.1 [Source:UniProtKB/Swiss-Prot;Acc:Q69P80]
OS11G0235200/LOC_Os11g12740	ORUFI11G07890		up	Nitrate transporter NTL1, putative, expressed [Source:UniProtKB/TrEMBL;Acc:Q53JI5]
OS06G0554800/LOC_Os06g36090	ORUFI06G19420	*ABC-2 type transporter*	down	
OS08G0156600/LOC_Os08g06010	ORUFI08G03760	*transporter, major facilitator family*	down	Os08g0156600 protein [Source:UniProtKB/TrEMBL;Acc:Q0J7X7]
OS12G0581600/LOC_Os12g39180	ORUFI12G18860	*NRAMP7*	down	Metal transporter Nramp6 [Source:UniProtKB/Swiss-Prot;Acc:Q2QN30]
**Other genes**				
OS01G0495701	ORUFI01G40710		up	Os01g0495701 protein [Source:UniProtKB/TrEMBL;Acc:A0A0P0V2W9]
OS01G0605100/LOC_Os01g42030	ORUFI01G24790	*BCS1*	up	BCS1 protein-like [Source:UniProtKB/TrEMBL;Acc:Q5ZDA1]
OS01G0608101	ORUFI01G25040		up	Os01g0608101 protein [Source:UniProtKB/TrEMBL;Acc:C7IWA4]
OS01G0655500/LOC_Os01g46720	ORUFI01G27970		up	Probable plastid-lipid-associated protein 14, chloroplastic [Source:Projected from Arabidopsis thaliana (AT5G53450) UniProtKB/Swiss-Prot;Acc:Q9LV04]
OS01G0775400/LOC_Os01g56810	ORUFI01G36000	*CKX5*	up	Cytokinin dehydrogenase 5 [Source:UniProtKB/Swiss-Prot;Acc:Q5ZAY9]
OS01G0878700/LOC_Os01g65670	ORUFI01G42590		up	Os01g0878700 Amino acid transporter, transmembrane domain containing protein [Source:UniProtKB/TrEMBL;Acc:Q5N9H2]
OS01G0952900/LOC_Os01g72360	ORUFI01G47550		up	Os01g0952900 protein [Source:UniProtKB/TrEMBL;Acc:Q0JFZ1]
OS02G0509500/LOC_Os02g30600	ORUFI02G18770		up	Os02g0509500 protein [Source:UniProtKB/TrEMBL;Acc:Q0E0Z7]
OS02G0579800/LOC_Os02g36940	ORUFI02G22960		up	Os02g0579800 protein [Source:UniProtKB/TrEMBL;Acc:Q6EP48]
OS02G0714600/LOC_Os02g48390	ORUFI02G31750		up	Ribose-phosphate pyrophosphokinase 4 [Source:UniProtKB/Swiss-Prot;Acc:Q6ZFT5]
OS02G0731900/LOC_Os02g49920	ORUFI02G33040		up	3-ketoacyl-CoA synthase [Source:UniProtKB/TrEMBL;Acc:A0A0N7KG16]
OS02G0744700/LOC_Os02g51070	ORUFI02G33990	*SSII-2*	up	Starch synthase, chloroplastic/amyloplastic [Source:UniProtKB/TrEMBL;Acc:Q0DXM0]
OS02G0791300/LOC_Os02g54870	ORUFI02G36990		up	Os02g0791300 protein [Source:UniProtKB/TrEMBL;Acc:Q6KAE4]
OS03G0161800/LOC_Os03g06620	ORUFI03G04320	*ARD2*	up	1,2-dihydroxy-3-keto-5-methylthiopentene dioxygenase 2 [Source:UniProtKB/Swiss-Prot;Acc:Q10RE5]
OS03G0327600/LOC_Os03g21040	ORUFI03G16490	*R40C1*	up	Ricin B-like lectin R40C1 [Source:UniProtKB/Swiss-Prot;Acc:Q10M12]
OS03G0401100/LOC_Os03g28300	ORUFI03G21290		up	Os03g0401100 protein [Source:UniProtKB/TrEMBL;Acc:Q10K10]
OS03G0431800/LOC_Os03g31730	ORUFI03G23070		up	Os03g0431800 protein [Source:UniProtKB/TrEMBL;Acc:A0A0P0VZP7]
OS03G0736900/LOC_Os03g52680	ORUFI03G34750		up	Os03g0736900 protein [Source:UniProtKB/TrEMBL;Acc:Q0DNS7]
OS01G0776600/LOC_Os01g56880	ORUFI01G36050		down	Purple acid phosphatase 10 [Source:Projected from Arabidopsis thaliana (AT2G16430) UniProtKB/Swiss-Prot;Acc:Q9SIV9]
OS01G0801600/LOC_Os01g58740	ORUFI01G37440		down	Probable glycerol-3-phosphate dehydrogenase [NAD(+)] 2, cytosolic [Source:UniProtKB/Swiss-Prot;Acc:Q8S2G5]
OS01G0814400/LOC_Os01g59900	ORUFI01G38230		down	lisH domain-containing protein C1711.05 [Source:UniProtKB/TrEMBL;Acc:A0A0P0V9N0]
OS01G0906000/LOC_Os01g67870	ORUFI01G44340		down	Os01g0906000 protein [Source:UniProtKB/TrEMBL;Acc:Q0JGT3]
OS01G0908200/LOC_Os01g68020	ORUFI01G44480	*BTBZ2 - Bric-a-Brac, Tramtrack, and Broad Complex BTB domain with TAZ zinc finger and Calmodulin-binding domains*	down	Os01g0908200 protein [Source:UniProtKB/TrEMBL;Acc:Q8L3R7]
OS01G0941800/LOC_Os01g71420	ORUFI01G47010		down	Probable inactive purple acid phosphatase 16 [Source:Projected from Arabidopsis thaliana (AT3G10150) UniProtKB/Swiss-Prot;Acc:Q9SR79]
OS02G0202200/LOC_Os02g10780	ORUFI03G26300	*SPX2*	down	SPX domain-containing protein 2 [Source:UniProtKB/Swiss-Prot;Acc:Q6Z784]
OS02G0327000/LOC_Os02g22130	ORUFI02G15000	*GAP1*	down	GTPase activating protein 1 [Source:UniProtKB/Swiss-Prot;Acc:Q6YWF1]
OS02G0514500/LOC_Os02g31030	ORUFI02G19040	*glycerophosphoryl diester phosphodiesterase family protein*	down	Os02g0514500 protein [Source:UniProtKB/TrEMBL;Acc:A0A0P0VJM6]
OS02G0542400/LOC_Os02g33770	ORUFI02G20760	*homeodomain*	down	Os02g0542400 protein [Source:UniProtKB/TrEMBL;Acc:Q6ESZ1]
OS04G0675000/LOC_Os04g57870	ORUFI04G31160		up	Os04g0675000 protein [Source:UniProtKB/TrEMBL;Acc:B9FDA0]
OS05G0482400/LOC_Os05g40384	ORUFI05G21860	*CYP714D1*	up	Cytochrome P450 714D1 [Source:UniProtKB/Swiss-Prot;Acc:Q5KQH7]
OS06G0114000/LOC_Os06g02380	ORUFI06G00900		up	Os06g0114000 protein [Source:UniProtKB/TrEMBL;Acc:Q9LWT6]
OS06G0486800/LOC_Os06g29180	ORUFI06G16260		up	Formate dehydrogenase, mitochondrial [Source:UniProtKB/TrEMBL;Acc:Q0DC43]
OS06G0549600/LOC_Os06g35650	ORUFI06G19050		up	Os06g0549600 protein [Source:UniProtKB/TrEMBL;Acc:Q5Z957]
OS06G0628032/LOC_Os06g42280	ORUFI06G23230		up	Os06g0628032 protein [Source:UniProtKB/TrEMBL;Acc:A0A0P0WZ43]
OS06G0702700/LOC_Os06g48960	ORUFI06G28520		up	Os06g0702700 protein [Source:UniProtKB/TrEMBL;Acc:Q5Z825]
OS08G0108700/LOC_Os08g01710	ORUFI08G00660		up	Os08g0108700 protein [Source:UniProtKB/TrEMBL;Acc:Q6ZC75]
OS08G0557200/LOC_Os08g44300	ORUFI08G25860	*RETROTRANSPOSON*	up	Calcineurin-like phosphoesterase family-like [Source:UniProtKB/TrEMBL;Acc:Q6ZJ14]
OS09G0129600/LOC_Os09g04339	ORUFI09G01420		up	Os09g0129600 protein [Source:UniProtKB/TrEMBL;Acc:A0A0P0XKA3]
OS09G0453800/LOC_Os09g28050	ORUFI09G13500		up	Aminotransferase [Source:UniProtKB/TrEMBL;Acc:Q67UZ0]
OS10G0195250/LOC_Os10g11889	ORUFI12G07700		up	Os10g0195250 protein [Source:UniProtKB/TrEMBL;Acc:B9G7W2]
OS11G0484000/LOC_Os11g29370	ORUFI11G14510		up	Probable bifunctional methylthioribulose-1-phosphate dehydratase/enolase-phosphatase E1 [Source:UniProtKB/Swiss-Prot;Acc:Q2R483]
OS12G0260500/LOC_Os12g16010	ORUFI12G08470	*sex determination protein tasselseed-2*	up	Os12g0260500 protein [Source:UniProtKB/TrEMBL;Acc:A0A0P0Y8Z9]
OS12G0589100/LOC_Os12g39860	ORUFI12G19290		up	Adenine phosphoribosyltransferase 1, putative, expressed [Source:UniProtKB/TrEMBL;Acc:Q2QMV8]
OS01G0142300/LOC_Os01g04920	ORUFI01G02770	*glycosyl transferase*	down	Os01g0142300 protein [Source:UniProtKB/TrEMBL;Acc:Q5ZBM2]
OS01G0227100/LOC_Os01g12710	ORUFI01G08790	*NYC1*	down	Probable chlorophyll(ide) b reductase NYC1, chloroplastic [Source:UniProtKB/Swiss-Prot;Acc:Q5N800]
OS01G0741900/LOC_Os01g53880	ORUFI01G33490	*IAA6*	down	Auxin-responsive protein IAA6 [Source:UniProtKB/Swiss-Prot;Acc:Q8LQ74]
OS02G0695600/LOC_Os02g46830	ORUFI02G30390		down	Os02g0695600 protein [Source:UniProtKB/TrEMBL;Acc:Q6YUQ1]
OS02G0704900/LOC_Os02g47600	ORUFI02G31040	*IPP*	down	Soluble inorganic pyrophosphatase [Source:UniProtKB/TrEMBL;Acc:B7E5N1]
OS02G0802700/LOC_Os02g55910	ORUFI02G37800	*MGD3*	down	Os02g0802700 protein [Source:UniProtKB/TrEMBL;Acc:A0A0P0VQS5]
OS03G0130400/LOC_Os03g03820	ORUFI03G02170		down	Probable adenylate kinase 1, chloroplastic [Source:UniProtKB/Swiss-Prot;Acc:Q10S93]
OS03G0214400/LOC_Os03g11560	ORUFI03G08360		down	Digalactosyldiacylglycerol synthase 2, putative, expressed [Source:UniProtKB/TrEMBL;Acc:Q10Q06]
OS03G0238600/LOC_Os03g13540	ORUFI03G10060		down	Purple acid phosphatase [Source:UniProtKB/TrEMBL;Acc:Q10PD0]
OS04G0110600/LOC_Os04g02000	ORUFI04G00600		down	Os04g0110600 protein [Source:UniProtKB/TrEMBL;Acc:Q0JFE3]
OS04G0438600/LOC_Os04g35790	ORUFI04G14680	*GLTP domain containing protein*	down	Os04g0438600 protein [Source:UniProtKB/TrEMBL;Acc:Q0JD08]
OS04G0480900/LOC_Os04g40490	ORUFI04G17670		down	OSJNBb0011N17.5 protein [Source:UniProtKB/TrEMBL;Acc:Q7XUQ7]
OS04G0508200/LOC_Os04g42920	ORUFI04G19660		down	Isocitrate dehydrogenase [NADP] [Source:UniProtKB/TrEMBL;Acc:Q0JBV4]
OS04G0627300/LOC_Os04g53560	ORUFI04G27820		down	Os04g0627300 protein [Source:UniProtKB/TrEMBL;Acc:B9FCM8]
OS04G0640600/LOC_Os04g54800	ORUFI04G28560	*shikimate kinase,*	down	Os04g0640600 protein [Source:UniProtKB/TrEMBL;Acc:A0A0P0WFI9]
OS04G0652700/LOC_Os04g55850	ORUFI04G29370	*nuclease PA3,*	down	Os04g0652700 protein [Source:UniProtKB/TrEMBL;Acc:B9FCW0]
OS05G0178300/LOC_Os05g08554	ORUFI05G05490	*OsCDT5*	down	Similar to Cadmium tolerant 1. (Os05t0178300-01) [Source: https://rapdb.dna.affrc.go.jp/tools/search/run?id=on&attr=desc&keyword=Os05g0178300]
OS05G0566400/LOC_Os05g49140	ORUFI05G27900		down	Mitogen-activated protein kinase [Source:UniProtKB/TrEMBL;Acc:Q0DFW7]
OS06G0115800/LOC_Os06g02540	ORUFI06G01060		down	Os06g0115800 protein [Source:UniProtKB/TrEMBL;Acc:Q0DF43]
OS06G0603600/LOC_Os06g40120	ORUFI06G21760	*SPX1*	down	SPX domain-containing protein 1 [Source:UniProtKB/Swiss-Prot;Acc:Q69XJ0]
OS07G0187700/LOC_Os07g09000	ORUFI07G05590	*WD domain, G-beta repeat domain containing protein*	down	Os07g0187700 protein [Source:UniProtKB/TrEMBL;Acc:Q6Z4F3]
OS07G0598200/LOC_Os07g40710	ORUFI07G22020	*circadian clock coupling factor-related*	down	Os07g0598200 protein [Source:UniProtKB/TrEMBL;Acc:Q6ZJE5]
OS07G0668700/LOC_Os07g47250	ORUFI07G26640	*lipase precursor,*	down	Os07g0668700 protein [Source:UniProtKB/TrEMBL;Acc:A0A0P0X9 × 0]
OS08G0118900/LOC_Os08g02540	ORUFI08G01360		down	Probable adenylate kinase 7, mitochondrial [Source:UniProtKB/Swiss-Prot;Acc:Q6ZJ48]
OS08G0245200/LOC_Os08g14760	ORUFI08G08590	*4CL1*	down	Probable 4-coumarate–CoA ligase 1 [Source:UniProtKB/Swiss-Prot;Acc:P17814]
OS08G0536000/LOC_Os08g42410	ORUFI08G24240		down	Pyruvate dehydrogenase E1 component subunit beta-1, mitochondrial [Source:UniProtKB/Swiss-Prot;Acc:Q6Z1G7]
OS09G0356000/LOC_Os09g19140	ORUFI09G07640	*senescence-induced receptor-like serine/threonine-protein kinase precursor*	down	Os09g0356000 protein [Source:UniProtKB/TrEMBL;Acc:A0A0P0XML9]
OS09G0569800/LOC_Os09g39620	ORUFI09G21810	*protein kinase family protein*	down	Os09g0569800 protein [Source:UniProtKB/TrEMBL;Acc:Q0IZH1]
OS10G0116800/LOC_Os10g02750	ORUFI10G00780	*OsPAP3b*	down	Purple acid phosphatase [Source:UniProtKB/TrEMBL;Acc:Q7XH73]
OS11G0126800/LOC_Os11g03290	ORUFI11G01490	*nucleoside-triphosphatase*	down	Os11g0126800 protein [Source:UniProtKB/TrEMBL;Acc:Q0IUZ4]
OS04G0523600/LOC_Os04g44240	ORUFI04G20630	*Glycosyltransferase*	up	Glycosyltransferase [Source:UniProtKB/TrEMBL;Acc:A0A0P0WCX8]

Genes were up- and down-regulated in common in both species.

**Figure 2:**
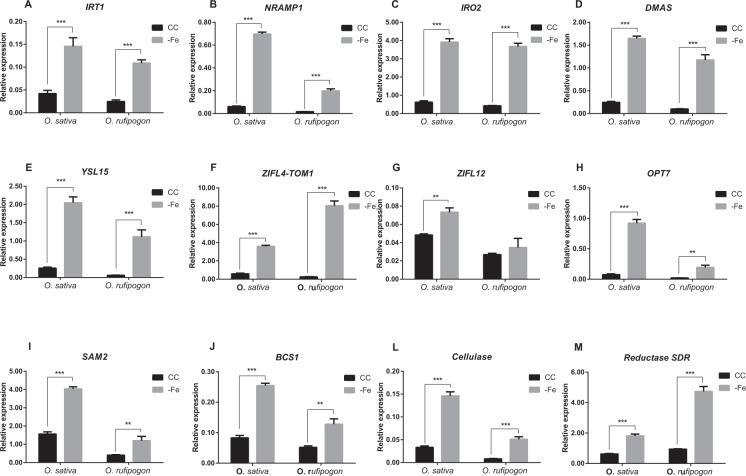
Expression analysis of selected genes ((**A**) *IRT1*; (**B**) *NRAMP1*; (**C**) *IRO2*; (**D**) *DMAS*; (**E**) *YSL15*; (**F**) *ZIFL4-TOM1*; (**G**) *ZIFL12*; (**H**) *OPT7*; (**I**) *SAM2*; (**J**) *BCS1*; (**L**) *Cellulase*; (**M**) *Reductase SDR*) in cultivated (*Oryza sativa*) and wild (*Oryza rufipogon*) rice. Relative expression levels (RT-qPCR, relative to *OsUBQ5* expression) of genes identified by RNAseq, in roots of plants submitted to control (CC) or Fe deficiency (−Fe) conditions for five days. Roots were collected from rice plants grown in non-aerated nutrient solution, at three-leaf stage on both conditions at the time of RNA extraction. Values are the averages of four samples (3 plants each) ± SE. Asterisks indicate statistical difference between plants grown under CC and −Fe conditions (Student *t*-test, **P-value < 0.01, ***P-value < 0.001).

In order to confirm the results obtained by RNAseq, we analyzed the expression level of two genes associated with Strategy I (*IRT1* and *NRAMP1*), five genes associated with Strategy II (*IRO2*, *DMAS*, *YSL15*, *ZIFL4-TOM1*, and *OPT7*), and *ZIFL12*, *SAM2*, mitochondrial chaperone *BCS1*, cellulase and one gene annotated as Reductase SDR Family (Supplementary Table 3). We selected genes that were either commonly regulated in both species, or were up-regulated only in *O. sativa* under −Fe condition. Differential expression was confirmed for all eight tested genes which were regulated similarly on both species according to the RNAseq data (*IRT1*, *NRAMP1*, *IRO2*, *DMAS*, *YSL15*, *ZIFL4-TOM1*, *OPT7*, *SAM2*, and mitochondrial chaperone *BCS1*; Fig. 2A–E,H–J). In addition, the genes *ZIFL4/TOM1*, cellulase and reductase SDR family, which were up-regulated in roots of *O. sativa* based on our RNAseq data, were shown to be also up-regulated in *O. rufipogon* (Fig. 2F,L,M). *ZIFL12* was up-regulated only in roots of *O. sativa* (Fig. 2G). Thus, we confirmed that genes associated with Fe acquisition from the soil in *O. sativa* are also up-regulated in *O. rufipogon* under Fe deficiency. These data further indicate that the two species use similar mechanisms for Fe uptake.

### Analysis of synteny and collinearity allow the inference of *OsIRT1*, *OsYSL15*, *OsIRO2*, and *OsNRAMP1* orthologs between *Oryza* species, maize and sorghum

**Figure 3:**
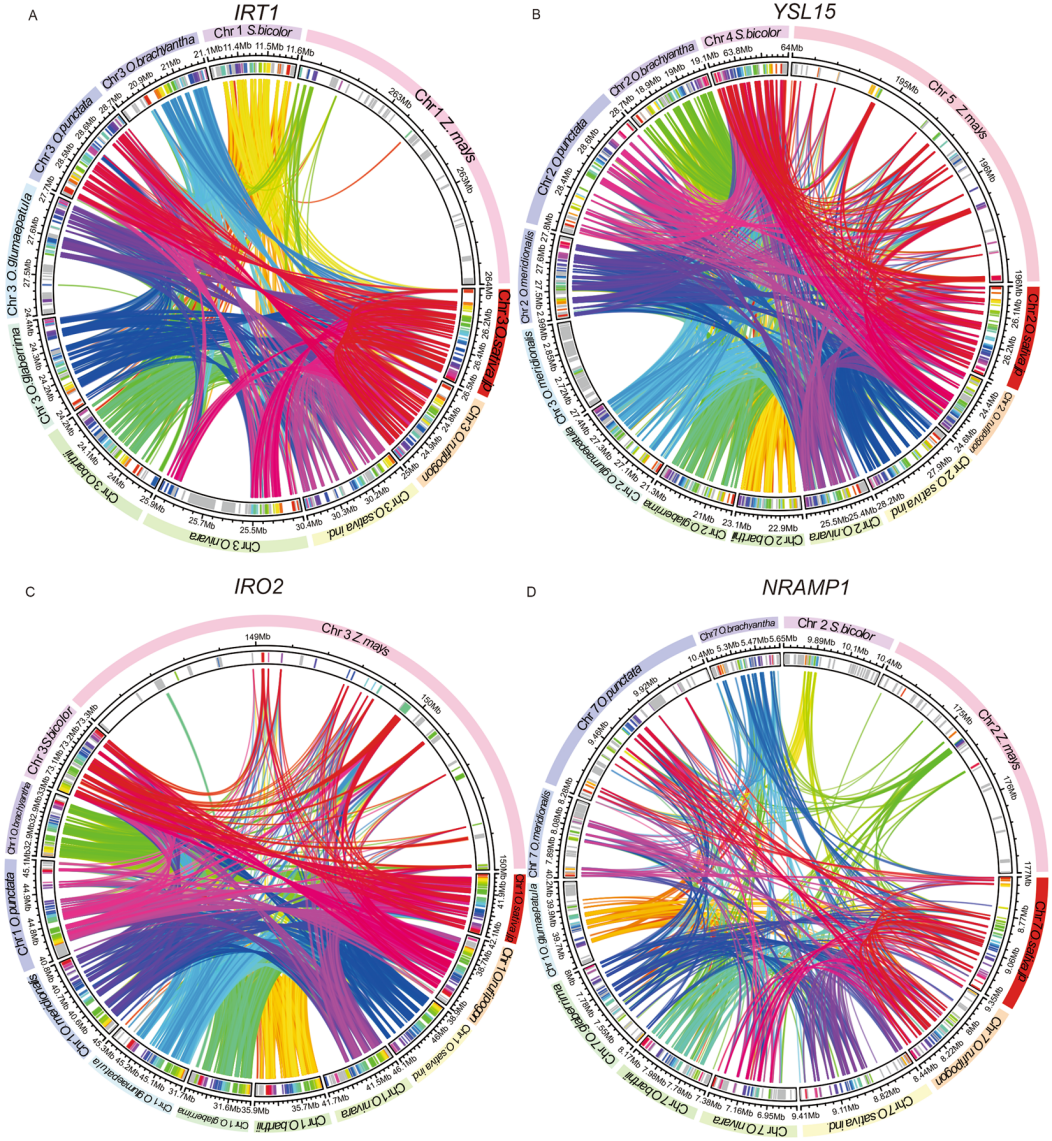
Synteny analysis of Fe deficiency-related genes in *Oryza spp*., maize and sorghum. Circular graphs displaying the results from the synteny analysis, performed through the MCSCanX tool, across the *Oryza* genus, *Zea mays*, and *Sorghum bicolor* gene orthologs. The analyses were performed on genomic segments containing 41 genes. Same-color ribbons connect syntenic genes from the same pair of genomic segments. Colored bars represent genes inside the genomic segments. The rainbow spectrum color bands inside the genomic segments are in reference to the 41 genes in the *O. sativa*’s chromosome segment containing the target gene (*YSL15*, *IRO2*, *NRAMP1* or *IRT1*) plus 20 genes up and downstream of it. A color-coded scheme is used to represent the genes inside the genomic segments as such: if a given gene is a potential homolog of any of the 41 afore mentioned set of reference genes, then it receives the same color of its homolog; otherwise, it is colored grey. (**A**) Synteny analysis for *IRT1*. (**B**) Synteny analysis for *YSL15*. (**C**) Synteny analysis for *IRO2*. (**D**) Synteny analysis for *NRAMP1*.

Based on the previous results, we hypothesized that wild rice species may also use the CS for Fe uptake. Since an expected characteristic of such strategy would be concomitant up-regulation of *OsYSL15* and *OsIRT1*, we decided to analyze the expression of these genes in other species of the *Oryza* genus and also in other grasses. We also included *IRO2*, a transcription factor involved in regulating the Strategy II genes in rice, and *NRAMP1*, an Fe transporter that is also up-regulated under Fe deficiency in cultivated rice, but has no clear function in Fe uptake yet. To identify the orthologous genes in nine species of the *Oryza* genus, maize and sorghum, we analyzed the syntenic relationships between their genomes using *O. sativa* ssp. *japonica* as a reference genome. We identified synteny in the *IRT1* locus for most species, with maize, *O. nivara* and *O. glumaepatula* having the least synteny compared to *O. sativa* (Fig. 3A). We found no *IRT1* ortholog in *O. meridionalis*. Scanning the *O. meridionalis* genome sequence, we found that the genome region that contains *IRT1* is likely missing from the current draft (Supplementary Fig. 4). We also identified syntenic genomic blocks for *YSL15* and *IRO2* (Fig. 3C) sequences in all genomes considered, except for *YSL15* in *O. meridionalis* (Fig. 3B). In this species, *YSL15* was found in chromosome 3, and not in chromosome 2, as in all other *Oryza* species. *OsYSL15* and *OsYSL2* are found *in tandem* in chromosome 2, an organization that is conserved in most *Oryza* species, but not in *O. meridionalis* (Fig. 3B). For *NRAMP1*, we identified synteny for most species considered (Fig. 3D).

Collinearity analyses showed the occurrence of inversions in the region containing *IRT1* when comparing *O. sativa* ssp. *japonica* with *O. nivara* and *O. glumaepatula* (Supplementary Fig. 5A). The segment containing the *YSL15* gene, located on chromosome 2 in *O. sativa*, tends to have genes in the same order and orientation as in *O. sativa* in most of the evaluated genomes, except in *O. meridionalis* and sorghum. When we consider *O. meridionalis*, the genomic block which is syntenic with *O. sativa* does not contain *YSL15* (Supplementary Fig. 5B). In this species, *YSL15* is located on chromosome 3. In relation to the transcription factor *IRO2*, the occurrence of collinearity with the *O. sativa* genome was observed in most of the analyzed species (Supplementary Fig. 5C). There was collinearity of the *NRAMP1* chromosomal region from *O. sativa* ssp. *japonica* with sorghum and maize only in the up and downstream regions, respectively (Supplementary Fig. 5D). These analyses allowed us to identify the most likely orthologs for *IRT1*, *YSL15*, *IRO2*, and *NRAMP1* in *Oryza* species, maize and sorghum.

### *Oryza* species with AA genome up-regulate genes used in the Combined Strategy under Fe deficiency conditions

**Figure 4:**
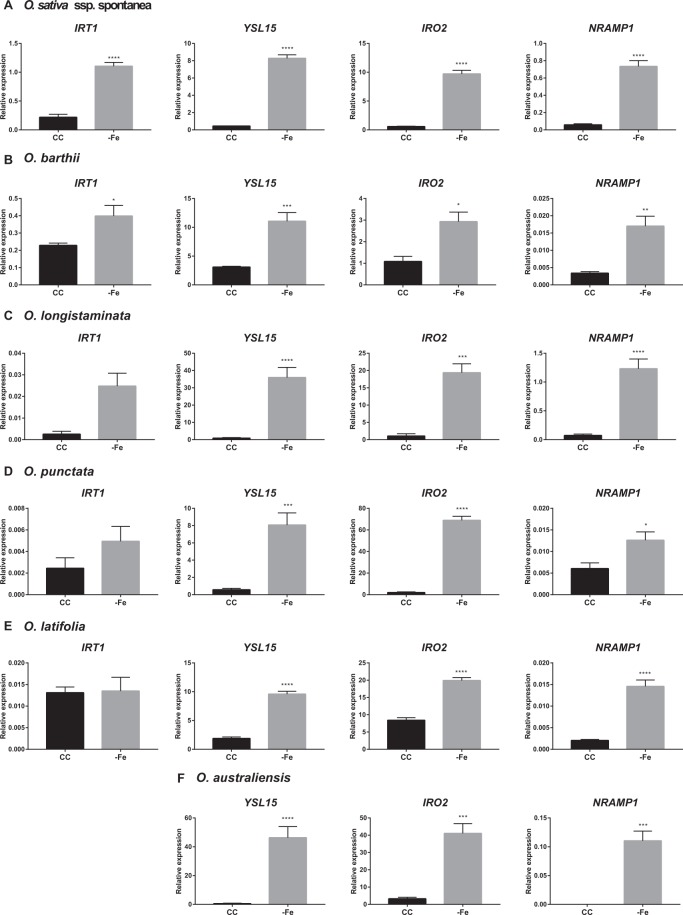
Expression analysis of Fe deficiency-related genes in (**A**) *Oryza sativa* ssp. *spontanea*; (**B**) *O. barthii*; (**C**) *O. longistaminata*; (**D**) *O. punctata*; (**E**) *O. latifolia*; (**F**) *O. australiensis*. Relative expression levels (RT-qPCR, relative to *OsUBQ5* expression) of selected genes (*IRT1*, *YSL15*, *IRO2*, and *NRAMP1*) in roots of plants submitted to control (CC) or Fe deficiency (−Fe) conditions for five days. Roots were collected from plants grown in non-aerated nutrient solution, at three-leaf stage on both conditions at the time of RNA extraction. Values are the averages of four samples (3 plants each) ± SE. Asterisks indicate statistical difference between plants grown under CC and −Fe conditions (Student *t*-test, *P-value < 0.05, **P-value < 0.01, ***P-value < 0.001, ****P-value < 0.0001).

To test whether the rice orthologous genes involved in the CS are up-regulated in other *Oryza* species, plants of *O. sativa* ssp. *spontanea* (AA genome type), *O. barthii* (AA), *O. longistaminata* (AA), *O. punctata* (BB), *O. latifolia* (CCDD) and *O. australiensis* (EE) were submitted to control or −Fe conditions for five days, and gene expression of *IRT1*, *YSL15*, *IRO2*, and *NRAMP1* was evaluated in roots. In *O. sativa* ssp. *spontanea* and *O. barthii*, expression of all four genes was up-regulated by −Fe (Fig. 4A,B). In *O. longistaminata*, *IRT1* expression seemed to be induced under −Fe, although not statistically significant (p = 0.067). However, the expression levels of *IRO2*, *YSL15*, and *NRAMP1* were up-regulated by −Fe in the same species (Fig. 4C). In *O. punctata* and *O. latifolia* roots, expression of *IRT1* was not significantly induced upon −Fe, while the transcript levels of *YSL15*, *IRO2*, and *NRAMP1* were up-regulated (Fig. 4D,E). *O. australiensis* also showed up-regulation of *YSL15*, *IRO2*, and *NRAMP1* (in this last, the expression was not detected in CC) in roots under −Fe, while *IRT1* expression was not detected, even under −Fe (Fig. 4F). These results suggest that it is possible that distinct species of *Oryza* genus evolved independently to Strategy II-exclusive or CS^20^, and indicate that the CS is common to other AA genome species besides *O. sativa*.

**Figure 5:**
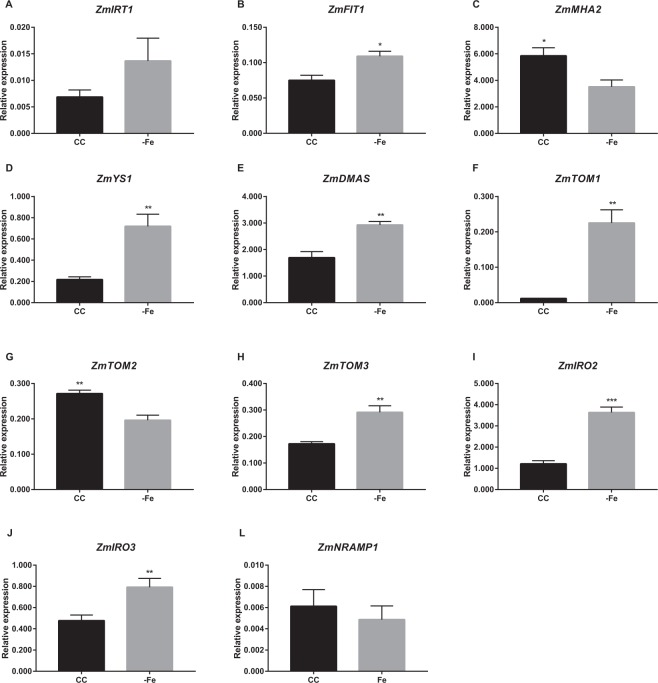
Expression analysis of Fe deficiency-related genes in *Zea mays*. Relative expression levels (RT-qPCR, relative to *ZmUBQ* expression) of selected genes ((**A**) *IRT1*; (**B**) *FIT1*; (**C**) *MHA2*; (**D**) *YS1*; (**E**) *DMAS*; (**F**) *TOM1*; (**G**) *TOM2*; (**H**) *TOM3*; (**I**) *IRO2*; (**J**) *IRO3*; (**L**) *NRAMP1*), in roots of plants submitted to control (CC) or Fe deficiency (−Fe) conditions for five days. Roots were collected from maize plants grown in aerated nutrient solution, at three-leaf stage on both conditions at the time of RNA extraction. Values are the averages of four samples (3 plants each) ± SE. Asterisks indicate statistical difference between plants grown under CC and −Fe conditions (Student *t*-test, *P-value < 0.05, **P-value < 0.01, ***P-value < 0.001).

**Figure 6:**
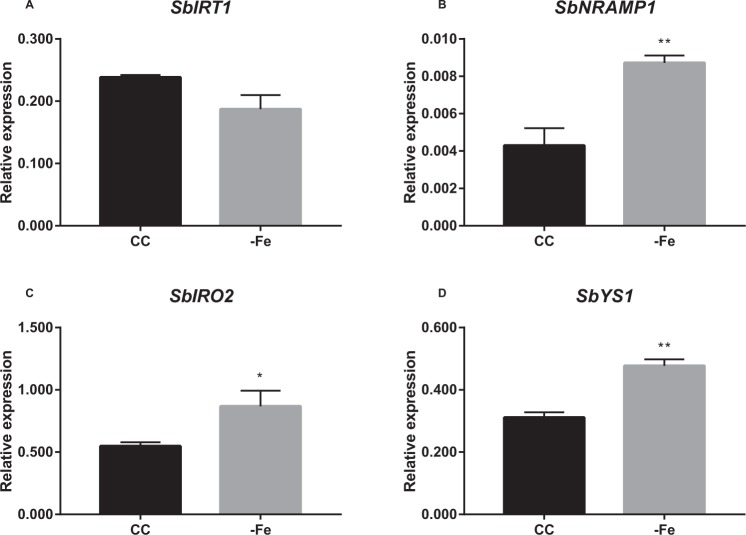
Expression analysis of Fe deficiency-related genes in *Sorghum bicolor*. Relative expression levels (RT-qPCR, relative to *SbACT* expression) of selected genes ((**A**) *IRT1*; (**B**) *NRAMP1*; (**C**) *IRO2*; (**D**) *YS1*), in roots of plants submitted to control (CC) or Fe-deficiency (−Fe) conditions for three days. Roots were collected from sorghum plants grown in aerated nutrient solution, at three-leaf stage on both conditions at the time of RNA extraction. Values are the averages of four samples (3 plants each) ± SE. Asterisks indicate statistical difference between plants grown under CC and −Fe conditions (Student *t*-test, *P-value < 0.05, **P-value < 0.01).

We also evaluated the expression of the same genes in both maize and sorghum roots under CC and −Fe conditions (Figs. 5 and 6). Chlorophyll quantification showed that maize plants are also at the early phase of Fe deficiency response after five days (Supplementary Fig. 6). Expression profiles of genes related to Strategy I (*ZmIRT1*^28^, *ZmFIT1*^29^ and *ZmMHA2*^29^) were evaluated. *ZmFIT1*, orthologous to the transcription factor *AtFIT*, which regulates the expression of *FRO2* and *IRT1* in Arabidopsis^30^, was up-regulated in roots under −Fe (Fig. 5B). *ZmMHA2*, similar to plasma membrane H^+^ATPase from Arabidopsis (MHA2)^29^, was down-regulated (Fig. 5C). *ZmIRT1* expression was not significantly affected by Fe deficiency (Fig. 5A). Genes related to Strategy II (*ZmYS1*, *ZmDMAS*, *ZmTOM1*, *ZmTOM2*, *ZmTOM3*, *ZmIRO2*, and *ZmIRO3*) were up-regulated in roots grown under −Fe condition compared to those cultivated in CC, except for *ZmTOM2*, as described^31^ (Fig. 5D–J). *ZmNRAMP1* expression was not regulated by Fe deficiency (Fig. 5L).

Sorghum plants showed decreased chlorophyll concentration after two days of treatment (Supplementary Fig. 7). *SbIRT1* was not regulated by Fe deficiency in roots, while the transcript levels of *SbNRAMP1*, *SbIRO2*, and *SbYS1* were up-regulated under −Fe compared to CC (Fig. 6A–D). These results suggest that both maize and sorghum do not induce *IRT1* expression under −Fe conditions, and thus may be Strategy II-exclusive plants.

## Discussion

Currently, there is little experimental evidence to support models of Fe deficiency response evolution in plants^20^. Evidence from *Chlamydomonas reinhardtii* and *Marchantia polymorpha* support the hypothesis that reduction-based Strategy I is ancestral in the plant lineage^32,33^. In this work, to add information to the evolution of Fe uptake strategies in Poaceae, we compared the root transcriptomic responses of *O. sativa* and *O. rufipogon* under −Fe. We choose *O. rufipogon* because this species is the wild progenitor of *O. sativa* ssp. *japonica*^34^. This is the first report of such comparison in the literature, and the first characterization of *O. rufipogon* Fe deficiency response. It is also the first RNAseq dataset for cultivated rice under −Fe, which allows us to confirm other studies using microarrays^35,36^. Our data also allowed us to propose a model for −Fe response evolution (see next sections).

We used physiological characterization to identify the early phase of −Fe response, which was established at five days for both *O. sativa* and *O. rufipogon* (Fig. 1). Transcriptomic data showed that both species have significant overlapping sets of regulated genes in roots upon −Fe treatment (Table 1 and Supplementary Fig. 2), although a large group of genes are exclusively regulated in one species (Supplementary Fig. 3). Among those regulated in both, we found genes linked to Fe homeostasis. HRZ1 and HRZ2, two hemerythrin domain-containing ubiquitin ligases that negatively regulate −Fe response through protein degradation^27^, are up-regulated in both species. HRZ1 and HRZ2 are involved in degradation of some components of the Fe uptake machinery, similar to the role of their Arabidopsis homolog BRUTUS^37^. Two bHLH transcription factors, *IRO2* and *IRO3*, were also up-regulated in roots of both species under −Fe. IRO2 is a positive regulator of Fe uptake genes, whereas IRO3 is a negative regulator, orthologous to Arabidopsis POPEYE^38^.

We found transporters that are associated with Fe homeostasis in cultivated rice also regulated in *O. rufipogon*. OsYSL9 was described as an Fe-chelate transporter involved in Fe distribution in developing grains, which is up-regulated by −Fe in roots^39^ (Table 1). *OsOPT7*, also described as an up-regulated gene in roots upon −Fe^40^, but with unknown transport substrate, was also up-regulated in *O. rufipogon* (Table 1). *ENA1*, described as a NA efflux transporter in rice, was also up-regulated in both species in our experiments^14^. Interestingly, we also found genes related to homeostasis of other metals, such as zinc (Zn, *ZIP4*^41^), copper (Cu; *COPT5.1*^42^) and metallothioneins (Table 1). Transporters such as *IRT1* are capable of transporting a variety of cations other than Fe, including cadmium (Cd), cobalt (Co), manganese (Mn) and Zn^10^, which can lead to accumulation of heavy metals such as Cd^43^ and Zn^44^ in plants when *IRT1* is highly expressed. Thus, up-regulation of transporters of other metals might be a consequence of the broad substrate specificity of IRT1-like transporters^45^ and accumulation of toxic levels of heavy metals. Moreover, we found the vacuolar iron transporter 2 (*VIT2*), which is up-regulated by Fe excess and down-regulated by −Fe, among the genes down-regulated in both species, corroborating previous data^46,47^. Finally, the uncharacterized *NRAMP7* transporter was also down-regulated in both species, suggesting a role for this transporter in Fe homeostasis.

Our GO analyses showed that Fe and methionine/S-adenosyl methionine-related processes are among the terms enriched in both species (Supplementary Fig. 2). Under −Fe, the methionine cycle feeds the biosynthesis of phytosiderophores in plants that rely on Strategy II to absorb Fe from the rhizosphere^19,35^. MAs are synthetized in a conserved pathway from S-adenosyl-L-methionine^13^, which is biosynthesized during the reaction of methionine with ATP by S-adenosyl-methionine synthase (SAMS)^48,49^. The pathway for biosynthesis of MA includes enzymatic reactions meditated by nicotianamine synthase (NAS1) which catalyzes the formation of NA. Next, nicotianamine transferase (NAAT1)^21^ converts NA into a 3′-keto intermediate, which is then reduced to deoxymugineic acid (DMA) by deoxymugineic acid synthase (DMAS)^13,40^. The synchronous expression of the methionine cycle-related genes was observed during the first 36 hours of −Fe in roots of rice^50^. Between the transcripts up-regulated only in *O. sativa*, we identified an aspartate aminotransferase (*OsIDI4*) which has been predicted to be the enzyme that catalyzes the conversion of 2-keto-4-methylthiobutyric acid to methionine by transamination, and is a candidate enzyme for the final step of the methionine cycle^51–53^. Up-regulation of genes involved in the biosynthesis of methionine and MA, like *SAM2*, *NAS1*, and *DMAS* were observed in *O. sativa* and *O. rufipogon* (Table 1). These results are indicative that both species responded to −Fe by increasing precursors and phytosiderophore synthesis for Fe uptake^54^.

We also found *ZIFL4/TOM1* up-regulated in roots of both species when exposed to −Fe. In rice, OsZIFL4/TOM1 was characterized as the transporter involved in DMA secretion to the rhizosphere, and thus a crucial player in Strategy II. DMA binds to Fe(III), generating the Fe(III)-phytosiderophore complex. Barley plants were also shown to use the ortholog HvTOM1 to perform this key step in phytosiderophore release^14^, while maize *ZmTOM1* was shown to be up-regulated by −Fe^31^. Thus, up-regulation of *ZIFL4/TOM1* in *O. rufipogon* fits well with what is expected in a plant acquiring Fe using Strategy II/chelation strategy. In agreement with that, we also found both *OsYSL15* and *OrYSL15* genes up-regulated in roots of Fe deficient plants. As YSL15 transporters are necessary for Fe(III)-phytosiderophore uptake into root cells, it is clear that a full Strategy II is being induced in both *O. sativa* and *O. rufipogon* under −Fe conditions.

Among the genes up-regulated by −Fe in both species, we found genes classically characterized as part of the Strategy I, similar to *IRT1*^10^ and *IRT2*^55^. *AtIRT1* encodes a high affinity Fe^2+^ transporter^10,18^ up-regulated in epidermis and exodermis of roots under −Fe. In rice, the most similar gene, *OsIRT1*, is induced by low Fe, and was able to complement yeast mutants defective in Fe transport^18^. It was shown that *OsIRT1* is part of the rice Fe regulon, being regulated along with Strategy II genes^36^. *OsIRT1* over-expression results in Fe, Zn, and Cd accumulation in rice tissues^56^. Based on these evidences, it was proposed that *OsIRT1* is involved in Fe uptake in cultivated rice, allowing rice roots to acquire Fe^2+^ from the soil. This would be an adaptation to paddy fields where rice is commonly cultivated, and where Fe^2+^ is much more abundant than Fe^3+^ ^18^.

Another gene found up-regulated in *O. sativa* and *O. rufipogon* was *NRAMP1*. In cultivated rice, OsNRAMP1 was characterized as an Fe^2+^, Mn^2+^, and Cd^2+^ transporter up-regulated by −Fe^57^. High *OsNRAMP1* expression was also linked to Cd^2+^ accumulation in rice cultivars^57^. A highly similar gene, *OsNRAMP5*, is also involved in Cd^2+^ accumulation, and is able to transport Fe^2+^ and Zn^2+^ ^58^. In Arabidopsis, the high-affinity metal transporter NRAMP1, which is closely related to *OsNRAMP1/OsNRAMP5*, is induced by −Fe, and was shown to be important for Fe transport under Fe sufficiency conditions, cooperating with *IRT1* to absorb Fe^+2^ from the rhizosphere^59^. However, to date, the physiological function of the rice ortholog *OsNRAMP1* is not clear. Thus, it is possible that rice plants use three transporters for primary Fe uptake from the soil under −Fe conditions (i.e., YSL15, IRT1, and NRAMP1), with OsNRAMP1 (or OsNRAMP5) having a role for Fe^2+^ uptake under Fe sufficient conditions. These hypotheses, however, need to be supported by additional evidence. Taken together, our data indicate that *O. rufipogon* also uses Strategy I-related genes such as *IRT1* and *NRAMP1* for Fe uptake, as observed in *O. sativa*.

After the first work showing that *O. sativa* is able to transport Fe^2+^ in addition to the ability to transport Fe(III)-phytosiderophore^18^, the current model for the evolution of Fe deficiency response was established, in which rice uses the CS as a recent adaptation to waterlogged soils^18,20^. Recently, we hypothesized that the underlying assumption that Fe^2+^ uptake by IRT1, considered as a new adaptation of cultivated rice, might not be true, and other *Poaceae* species might also use IRT1 for Fe uptake, which would mean the IRT1-based CS has a common, more ancient origin within the family or the *Oryza* genus. In order to test this hypothesis in wild *Oryza* species, maize and sorghum, we analyzed the expression of the orthologous genes of *YSL15* and *IRT1*, which control Fe^3+^ and Fe^2+^ uptake, respectively, in plants under −Fe. We also included *IRO2*, a positive regulator of the Strategy II genes in rice; and *NRAMP1*, which is part of the Fe regulon in rice and may also be involved in Fe uptake. Expression of the orthologous genes (Fig. 3 and Supplementary Fig. 5) encoding each of these proteins in all species was analyzed. We assumed that concerted up-regulation of both *YSL15* and *IRT1* was indicative of CS use in a given species. *NRAMP1* up-regulation was considered as further indication of a conserved role for this gene in −Fe response. Importantly, our approach aims at identifying a common origin of the CS in *Poaceae*, based on the IRT1 orthologs in these species. It is possible that independent CS strategies have evolved in this family, which could be based on other transporters that perform the same activity (i.e., NRAMP1 or IRT2).

*O. sativa* ssp. *spontanea* (AA genome) is the weedy rice variety, and is cultivated in flooded soil along with cultivated rice, being widely distributed in South and South-east Asia, South and North America, and southern Europe^60^. *O. barthii* and *O. longistaminata* (both AA genomes) are species cultivated in seasonally dry habitats. These three species are representative of the *O. sativa* complex, which include AA genome species, the closest ones to cultivated rice in the *Oryza* genus^61,62^. In these three species, we observed increased expression of both *IRT1* and *YSL15*. *NRAMP1* and *IRO2* were also up-regulated (Fig. 4A–C). We also tested three species outside the *O. sativa* complex: *O. punctata* (BB genome), *O. latifolia* (CCDD genome), and *O. australiensis* (EE genome), all from the *O. officinalis* complex. These species are cultivated in seasonally dry habitats^61^. Interestingly, we observed up-regulation of *YSL15*, *NRAMP1*, and *IRO2*, but not of *IRT1* (Fig. 4D–F). These results suggest that species from the *O. officinalis* complex do not use IRT1 for Fe uptake under low Fe conditions, while species from the *O. sativa* complex up-regulate *IRT1* under the same conditions.

We also evaluated the expression of the same genes in maize and sorghum, two *Poaceae* species outside the *Oryza* genus that serve as out groups to test our hypothesis for a common origin of an IRT1-based CS in *Poaceae*. Even though the Fe uptake mechanism has not been as well characterized in maize and sorghum as in *O. sativa*, there has been some discussion whether maize can use a partial Strategy I^29,31,63^. We observed that *ZmIRT1* expression was not significantly induced in maize roots exposed to −Fe, although there seems to be a trend for higher expression under iron deficiency (Fig. 5A). Nozoye *et al*.^31^ and Li *et al*.^28^ found *ZmIRT1* up-regulated under similar conditions. *ZmYS1*, as expected, was clearly up-regulated (Fig. 5D). We also tested other candidate genes likely involved in −Fe regulation in maize already described in the literature to confirm that maize plants are responding properly to −Fe conditions^29^ (Fig. 5). When we tested the likely orthologs of our candidate genes in sorghum, we observed that *SbIRT1* was not up-regulated, while *SbYS1* was (Fig. 6D). Interestingly, *ZmNRAMP1* was not regulated by −Fe, whereas *SbNRAMP1* was. These results suggest that sorghum plants do not use the CS, whereas maize may use ZmIRT1 as an Fe^2+^ transporter upon Fe deficiency, but without a strong, consistent up-regulation. Alternatively, since we used a different genotype than Nozoye *et al*., *ZmIRT1* up-regulation might be genotype specific, as observed for barley *HvIRT1*^64^. Thus, as already suggested^20^, the use of CS might vary in *Poaceae* species outside the *Oryza* genus or even in genotypes within the genus, which may be linked to local adaptation to specific environments where each genotype is found. Considering the possibility that maize also uses the CS based on the *IRT1* ortholog, our data suggest that maize CS is not homologous to what is observed in the AA *Oryza* group, which would indicate an independent/convergent evolution in the use of *IRT1*, together with the shared chelation strategy. Altogether, our data suggest a restricted up-regulation of *IRT1* and *YSL15* within the AA genome group of the *Oryza* genus, resembling the *O. sativa* CS. Moreover, *NRAMP1* does not seem to have a conserved function in all *Poaceae* species analyzed.

## Conclusion

**Figure 7:**
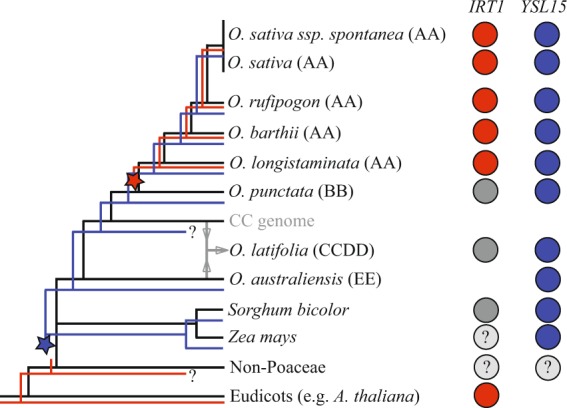
Model for the evolution of rice Combined Strategy (CS) of Fe uptake in the *Poaceae* family. Red and blue lines represent *Oryza* species using the CS for Fe uptake, showed by *IRT1* up-regulation (partial Strategy I) and *YSL15* up-regulation (Strategy II) under Fe deficiency. Only red or blue lines represent plants exclusively using Strategy I or Strategy II, respectively. Red and blue circles represent species in which *IRT1* and *YSL15* are expressed in roots under −Fe, respectively. Dark grey circles represent species in which *IRT1* expression is not induced under −Fe. Light grey circles represent species in which regulation of key genes is unclear or not yet known. The red star represents the split of *O. sativa* complex within the *Oryza* genus, in which *IRT1* is up-regulated under −Fe, and the CS is conserved. The blue star represents the split between *Poaceae* last common ancestor and other monocots lineages.

In conclusion, our data suggest that Strategy II for Fe uptake, represented by *YSL15* up-regulation under −Fe, is conserved in the *Poaceae* family, being an evolutionary novelty after the split between *Poaceae* last common ancestor and other monocots lineages (Fig. 7). Partial Strategy I for Fe uptake based on up-regulation of *IRT1* orthologs under −Fe is conserved only within the *O. sativa* complex containing the AA genome group. Therefore, the species with AA genome share a common origin for the *IRT1* up-regulation, which is an evolutionary novelty that arose after the split of the AA lineage from the last common ancestor with the BB genome lineage (Fig. 7). We also propose that the CS is not exclusive of cultivated rice, but rather common in wild species closely related to *O. sativa*, and likely an adaptation to flooded soils that preceded speciation within the *O. sativa* complex. This improves our knowledge about the evolution of Fe uptake mechanisms in plants, and especially in the *Poaceae* family.

## Methods

### Plant material and treatments

Seeds of *Oryza sativa* L. (Nipponbare cultivar) and *O. rufipogon* (BRA 00004909-8 accession from EMBRAPA Rice & Beans) were germinated for four days in Petri dishes with filter paper soaked in distilled water at 28 °C (two days in the dark and two days in the light, 40 µmol.m^−2^.s^−1^). After germination, plants were transferred to vermiculite and cultivated for fifteen days in distilled water. Then, plants were transferred to plastic pots with 0.5 L of nutrient solution (five plants per pot) containing 700 μM K_2_SO_4_, 100 μM KCl, 100 μM KH_2_PO_4_, 2 mM Ca(NO_3_)_2_, 500 μM MgSO_4_, 10 μM H_3_BO_3_, 0.5 μM MnSO_4_, 0.5 μM ZnSO_4_, 0.2 μM CuSO_4_, 0.01 μM (NH_4_)_6_Mo_7_O_24_, and 100 μM Fe^+3^-EDTA. After seven days of acclimation, plants were transferred to control condition (CC; containing 100 μM Fe^+3^-EDTA) or to Fe deficiency treatment (−Fe; no iron added) for one, three, five, seven and nine days. To evaluate the response to Fe deficiency in the *Oryza* genus, seeds from the species *O. australiensis* (IRGC 86526), *O. barthii* (IRGC 86524), *O. latifolia* (IRGC 103808), *O. longistaminata* (IRGC 101254), *O. punctata* (IRGC 88825), and *O. sativa* ssp. *spontanea* (IRGC 86719) were cultivated. Before germination, seeds were submitted to 50 °C for seven days to break dormancy, according to instructions provided by the International Rice Research Institute (IRRI). After, seeds were germinated and seedlings were cultivated without aeration as described above.

Maize (genotype B73)^65^ and sorghum (genotype BTx623)^66^ were germinated in vermiculite and nutrient solution (as described above) for fifteen days, and transferred to pots with 6 L of hydroponic solution (30 plants per pot) aerated by air pumps. Plants were then transferred to control conditions (CC, 100 μM Fe^+3^-EDTA) or to Fe deficiency (−Fe; no iron added) treatment. The pH of the nutrient solutions was adjusted to 5.4. Plants were grown in a growth room at 26 °C ± 1 °C under photoperiod of 16 h/8 h light/dark (150 μmol.m^−2^.s^−1^). Solutions were replaced twice a week.

### Chlorophyll quantification

Samples from the two youngest fully expanded leaves (about 100 mg) from plants submitted to CC or −Fe conditions were collected (n = 4, each sample consisting of three pooled plants), frozen in liquid nitrogen and stored at −80 °C. Chlorophyll extraction was performed in 85% (v/v) acetone. Chlorophyll *a* and *b* were quantified by measuring absorbance at 643 and 663 nm, and the concentrations calculated according to Ross^67^. Measurements of relative leaf chlorophyll level were performed with Soil Plant Analysis Development (SPAD-502, Minolta, Japan; n = 10).

### RNA extraction and transcriptome analyses by RNAseq

Rice roots samples were harvested from plants grown in CC and from plants submitted to −Fe treatment for one, three, five, seven and nine days. Total RNA was extracted using Concert Plant RNA Reagent (Invitrogen^®^, Carlsbad, USA), according to the manufacturer’s instructions, quantified by Nanodrop and treated with DNAse I (Invitrogen^®^, Carlsbad, USA). Approximately 20 µg of total RNA was used to high-throughput cDNA sequencing by Illumina HiSeq. 2000 technology (Fasteris SA, Chemin du Pont-du-Centenaire, Switzerland – http://www.fasteris.com/). RNAs derived from three biological replicates were pooled to generate each library. A total of eight libraries were constructed, from samples harvested five days after the onset of treatments: two libraries from roots of each *O. sativa* and *O. rufipogon* grown in CC and two libraries from roots grown in −Fe for each species. The cDNA libraries were prepared according to Illumina’s protocols, as described^68^. After sequencing, read quality was checked by FastQC (https://www.bioinformatics.babraham.ac.uk/projects/fastqc/), and all low quality reads (PHRED value < 30) were removed. Adapter sequences were trimmed using Trim Galore (https://www.bioinformatics.babraham.ac.uk/projects/trim_galore/) and the first twelve nucleotides were removed from the 5′ end. The abundance of each transcript was estimated using Kallisto^69^. Kallisto is a pseudoalignment RNAseq quantification method, in which the reads are pseudoaligned to a reference transcriptome, producing a list of transcripts that are compatible with each read. Kallisto does not assign each read to a physical position on a reference genome. Instead, it measures how likely the relative abundance of a transcript given a certain library dataset is. For transcripts proportions quantification, Kallisto uses an expectation-maximization algorithm to optimize a likelihood function. This optimization process will output a set of parameters that quantify the proportion of each transcript. Transcripts from different species or from different treatments were considered differentially expressed when the False Discovery Rate (FDR) < 0.05, according to the methodology proposed by Pimentel *et al*.^70^. Differentially expressed transcript annotations were downloaded from Ensembl plants database using the R package biomaRt^71^. The data is publicly available through the GEO database with accession number GSE131238.

### Gene ontology (GO) terms enrichment analysis

Comparison of differentially expressed genes in CC or −Fe conditions for *O. sativa* and *O. rufipogon* datasets was performed to find enriched Gene Ontology (GO) terms. The enrichment analysis was performed using topGO^72^. We used Fisher’s Exact Test and the GO terms with p < 0.05 were considered enriched.

### Gene expression analysis by RT-qPCR

Total RNA was extracted from roots of plants from eight species of the *Oryza* genus, maize and sorghum, submitted to CC or −Fe conditions for five days. After extraction with Concert Plant RNA Reagent (Invitrogen^®^, Carlsbad, USA), according to the manufacturer’s instructions, quantification was performed using Nanodrop^®^ (Thermo Fisher Scientific, Waltham, USA). Total RNA was treated with DNAse I (Invitrogen^®^, Carlsbad, USA), and first-strand cDNA synthesis was performed with OligodT and reverse transcriptase M-MLV (Invitrogen^®^, Carlsbad, USA) using 1 µg of DNAse-treated RNA. RT-qPCRs were carried out in a StepOne Real-Time Cycler (Applied Biosystems, Foster City, USA). For all species from the *Oryza* genus, genomic and coding sequences of selected genes were aligned using DiAlign Local multiple alignment (http://www.genomatix.de/cgi-bin/dialign/dialign.pl) to find conserved regions. Primers that could amplify the same region of each gene in all species with available genomes were designed. All primers (listed in Supplementary Table 3) were designed to amplify 100–200 bp and to have similar Tm values (60 °C ± 1 °C). Reactions settings were composed of an initial denaturation step of 5 min at 94 °C, followed by 40 cycles of 10 s at 94 °C, 15 s at 60 °C, 15 s at 72 °C and 40 s at 60 °C (fluorescence data collection). Samples were held for 2 min at 40 °C for annealing of the amplified products and then heated from 55 to 99 °C with a ramp of 0.1 °C/s to produce the denaturation curve of the amplified products. RT-qPCRs were carried out in 20 µL final volume composed of 10 µL of cDNA sample diluted 100 times, 2 µL of 10 × PCR buffer, 1.2 µL of 50 mM MgCl_2_, 0.2 µL of 10 mM dNTPs, 0.4 µL of each primer pair (10 µM), 3.82 µL of water, 2 µL of SYBR green (1:10,000 Molecular Probe), and 0.05 µL of Platinum Taq DNA Polymerase (5 U µL^−1^, Invitrogen, Carlsbad, CA, USA). Data were analyzed using the Pfaffl method^73^. The PCR efficiency from the exponential phase was calculated for each individual amplification plot using the LinReg software^74^. Since the method using LinReg allows for assumption-free calculation of PCR efficiency for each PCR reaction (all technical and biological replicates), we used efficiency averages in each PCR run for calculation. PCR efficiencies are shown in Supplementary Table 3. We used four biological replicates composed by roots from three plants each, and three technical replicates.

### Search for synteny and analysis of regions with similarities and/or differences between the genomes of species from the *Oryza* genus, maize, and sorghum

Whole genome and proteome sequences of nine species from the *Oryza* genus (*O. meridionalis*, *O. brachyantha*, *O. barthii*, *O. punctata*, *O. glumaepatula*, *O. nivara*, *O. glaberrima*, *O. sativa* ssp. *indica*, *O. sativa* ssp. *japonica*, and *O. rufipogon*), maize (genotype B73) and sorghum (genotype BTx623) were retrieved from Ensembl (http://ensembl.gramene.org/). The protein amino acid sequences of OsNRAMP1, OsIRT1, OsIRO2, and OsYSL15 were used as queries on a batch BLASTp^75^ search against the complete sets of annotated proteins for all species. For any queried protein X on each targeted species Y, the hit displaying the lowest E-value was selected as the best candidate for being the X homologous on species Y. This resulted in four groups composed by different sets of homologous proteins across the considered species. For each of these groups, synteny analysis was carried out separately as follows. For each gene that encodes a protein in the homologous set, we took a total of 20 genes upstream and 20 genes downstream from its locus. Next, in order to infer synteny degree among these genomic blocks, we used the McScanX software, following the methodology proposed on the original work^76^. The pairs of genes displaying an E-value < 10e^−10^ for the expected number of collinear blocks were considered to be in syntenic positions. Circular graphs displaying synteny relationships among the genomic blocks were built with the R package circlize^77^.

### Statistical analysis

Mean values were compared by the Student’s *t* test (p ≤ 0.05) using the GraphPad Prism 7 (GraphPad Software) for Windows.

## Supplementary information

Supplementary Material

Supplementary Dataset 1

Supplementary Dataset 2

## Data Availability

Authors declare that all data and materials used in this work are available to others upon request.
