# Supplementary material for: The Combined Strategy for iron uptake is not exclusive to domesticated rice (Oryza sativa)
Source: Sci Rep. 2019 Nov 6;9:16144. doi: 10.1038/s41598-019-52502-0 (PMC6834603; doi:10.1038/s41598-019-52502-0)

The Combined Strategy for iron uptake is not exclusive to domesticated rice (*Oryza sativa*)

Andriele Wairich[1], Ben Hur Neves de Oliveira[1], Ezequiel Barth Arend[2], Guilherme Leitão Duarte[1], Lucas Roani Ponte[3], Raul Antonio Sperotto[4], Felipe Klein Ricachenevsky[*1,3], Janette Palma Fett[*1]

Supplementary Material

Supplementary Table Legends

Supplementary Table 1. Full transcriptome data sets revealed by RNAseq in roots of *Oryza sativa* after five days under Fe-deficiency.

Supplementary Table 2. Full transcriptome data sets revealed by RNAseq in roots of *Oryza rufipogon* after five days under Fe-deficiency.

Supplementary Table 3. Gene-specific PCR primers used for RT-qPCR and PCR efficiencies.

Supplementary Table 3. Gene-specific PCR primers used for RT-qPCR.

Locus	Gene name	Forward Primer 5’ → 3’	Reverse Primer 5’ → 3’	PCR amplification efficiencies	
***Oryza sativa*/ *O. rufipogon*[A]**				**Eff target gene / endogenous *O. sativa***	**Eff target gene / endogenous *O.rufipogon***
OS01G0952800/ORUFI01G47560	*IRO2*	CGGATTTGGGAACAGGACA	GTTCCTGACGACTTTCTCCA	1.939027/1.886871	1.931052/1.86818
OS03G0667500/ORUFI03G29910	*IRT1*	ACTGGTGCCCATTCTGC	GCGAGGATGGGGATGG	1.877963/1.882419	1.912471/1.936508
OS01G0605100/ORUFI01G24790	*BCS1*	CTGGAACTCAACCCTTCGA	CCTCTGAAGGATTGGATTTGG	1.917836/1.889704	1.937038/1.906191
OS10G0370500	Cellulase	CACCCTGAGGTCGAACG	GGCGAACCGGACGAG	1.838172/1.862471	1.848612/1.860437
OS01G0323600/ORUFI01G14960	S-adenosylmethionine synthetase 2	ACGCCACCTTGCTGTC	GACGTTCCTCTTCACCTCC	1.888845/1.936181	1.886621/1.891841
OS07G0258400/ORUFI07G09420	*NRAMP1*	CATGCTGCTCTACGTCGTC	CAGCTCACGACGAGACAC	1.946169/1.886966	1.957729/1.953282
OS03G0751100/ORUFI03G35800	*OPT7*	AGTGTGAAGGCGCCG	ATCTTCTTCTTCGCGAGCTT	1.796674/1.825561	1.799743/1.816699
OS11G0134900	*ZIFL4-TOM1*	TGTGATTGAATTAATTGGACTTGC	GGGGTGCTATTCCAGCTTCT	1.935136/1.866774	1.93262/1.956244
OS03G0237100/ORUFI03G09980	*DMAS*	CCTGGACATCGTCGGAT	GTCGTCGAGCGACTTGTAG	1.883525/1.911072	1.892687/1.898705
OS09G0133200	*Reductase SDR Family*	GAGCTTATTGACAGGAGCACA	AGCAACAACAATAGTTTCAGCTG	1.944826/1.909023	1.899864/1.93218
OS02G0650300/ ORUFI02G27480	*YSL15*	GGTGCGGGGATGATTTG	CCATACAAACTTGTCATGCTG	1.949223/1.902821	1.925499/1.871946
OS12G0133100	*ZIFL12*	CCCAAACTGTTGAAGCTTTGG	GGACATCAAGGGCCAATTTC	1.894337/1.888208	1.884827/1.885545
OS01G0328400	*UBQ5*	ACCACTTCGACCGCCACTACT	ACGCCTAAGCCTGCTGGTT	-	-
Zea mays				***Eff target gene***	***Eff endogenous gene***
Zm00001d033446	*IRT1*	GCTTCTACAACCGGAGGAAG	GTCCTCGGGCTTGTCAG	1.940012	1.909559
Zm00001d017429	*YS1*	GTCTGGGAGAAGGTGAACAG	GGCCAGAGCGAGAATGG	1.894495	1.892154
Zm00001d028360	*DMAS*	GAGCTTCGACGAGGGG	GTGCTCGGAGACGTAGC	1.920767	1.879584
Zm00001d041111	*TOM1*	CTTTCCTTGTGGACCGTAAGT	ACAAATGAACATACCTGCAATCG	1.917432	1.861516

Zm00001d052434	*TOM2*	GACCCTACACAAGCACAGA	GGAGGGAGAAGACGCAGTA	1.865249	1.861516
Zm00001d005001	*TOM3*	GCCCACCAGAGTGACTAC	CTGAATAAGCCATATCATGGAAGG	1.897926	1.861516
Zm00001d042062	*IRO2*	GCCTCAACGACACGGAG	CCCAAAGGTGGAGTAGGTT	1.843356	1.892154
Zm00001d029339	*IRO3*	CTTTCCAGAAGTGGCATCTG	GCTTGTGCTGCTACTGC	1.866295	1.905458
Zm00001d053834	*UBQ*	CGGCAAGCAGCTGGAG	GCGCAGCACCAGGTG	-	-
Zm00001d025205	*FIT*	CCGAACGGCGGGTTATT	CACTGCTGGTAGTGGTCG	1.846875	1.879584
Zm00001d002006	*MHA2*	GTTCCTTGGGTTCATGTGGA	GGAGTTGATAACCAAGAGCACG	1.865067	1.905458
Zm00001d005479	*NRAMP1*	CTCATTGGCATTGTCGTGTT	CACGAACTTGATGGTCTCC	1.862461	1.768098
Sorghum bicolor				***Eff target gene***	***Eff endogenous gene***
SORBI_001G142900	*IRT1*	GACCGCAACCTCTTCGT	GGTGGTGAAGGAGAAATCGG	1.958446	1.958171
SORBI_003G428800	*IRO2*	CGGAGATCATGGTTCAGGT	CTTGTTCTCGAAGGTGGAGT	1.948199	1.913552
SORBI_002G095600	*NRAMP1*	GCATCATCGTGTTCCCTCTTAT	CATCGGCAACCTTTGATTTGTC	1.924862	1.914071
SORBI_004G299500	*YS1*	GAAGTACGCGAGGTTTGTG	CAGCCTCCTTCTTGTTCATC	1.913064	1.924862
SORBI_3001G112600	*ACT1*	GGCAACATCGTCCTCTCT	CCTCCAATCCAGACACTGT	-	-

Genes for which *O. rufipogon* loci is not shown were identified as differentially expressed by RNAseq only in *O. sativa.*

[A] *IRT1*, *NRAMP1*, *IRO2*, and *YSL15* primers are the same used for all species.

Supplementary Figure 1. Transcriptomic analysis of *Oryza sativa* and *Oryza rufipogon* roots from plants grown in control (CC) and Fe deficiency (-Fe) conditions for five days. Differential gene expression analysis of RNAseq from CC versus -Fe for (A) *O. sativa* and (B) *O. rufipogon*. Genes identified as differentially expressed by Kallisto (FDR < 0.05) are represented by red (up-regulated) and blue (down-regulated) lines. The *y*-axis represents a dendrogram from hierarchical clustering presented as transcripts per million reads (TPM). CC1: library 1 in control condition; CC2: library 2 in control condition; -Fe1: library 1 in iron deficiency condition; -Fe2: library 2 in iron deficiency condition.

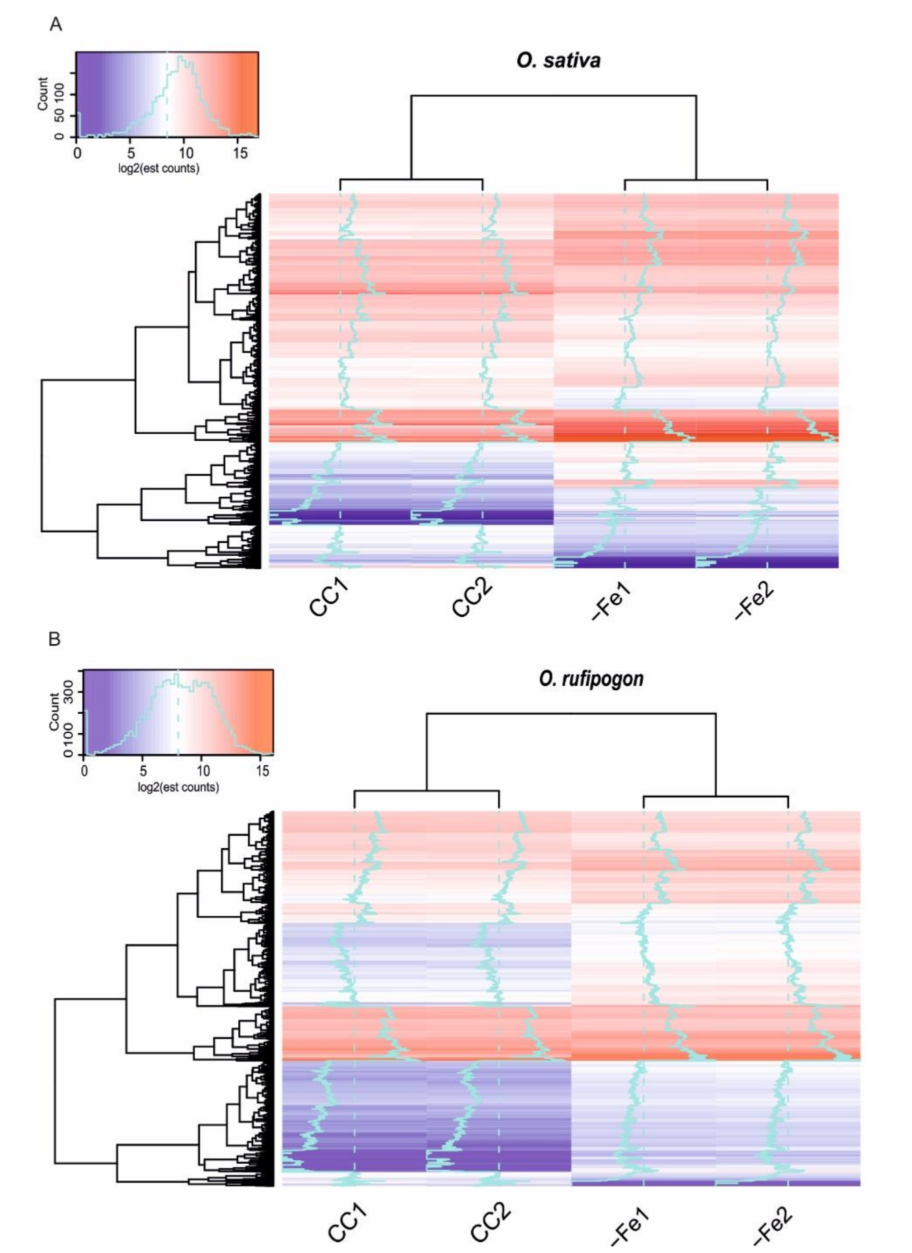

Supplementary Figure 2. Shared Gene Ontology (GO) terms in the response to Fe deficiency between cultivated (*Oryza sativa*) and wild (*Oryza rufipogon*) rice. The figure presents the terms regulated in common and with high level of significance for both species. (A) Up-regulated processes. (B) Down-regulated processes. Blue columns refer to *O. sativa*. Orange columns refer to *O. rufipogon.*

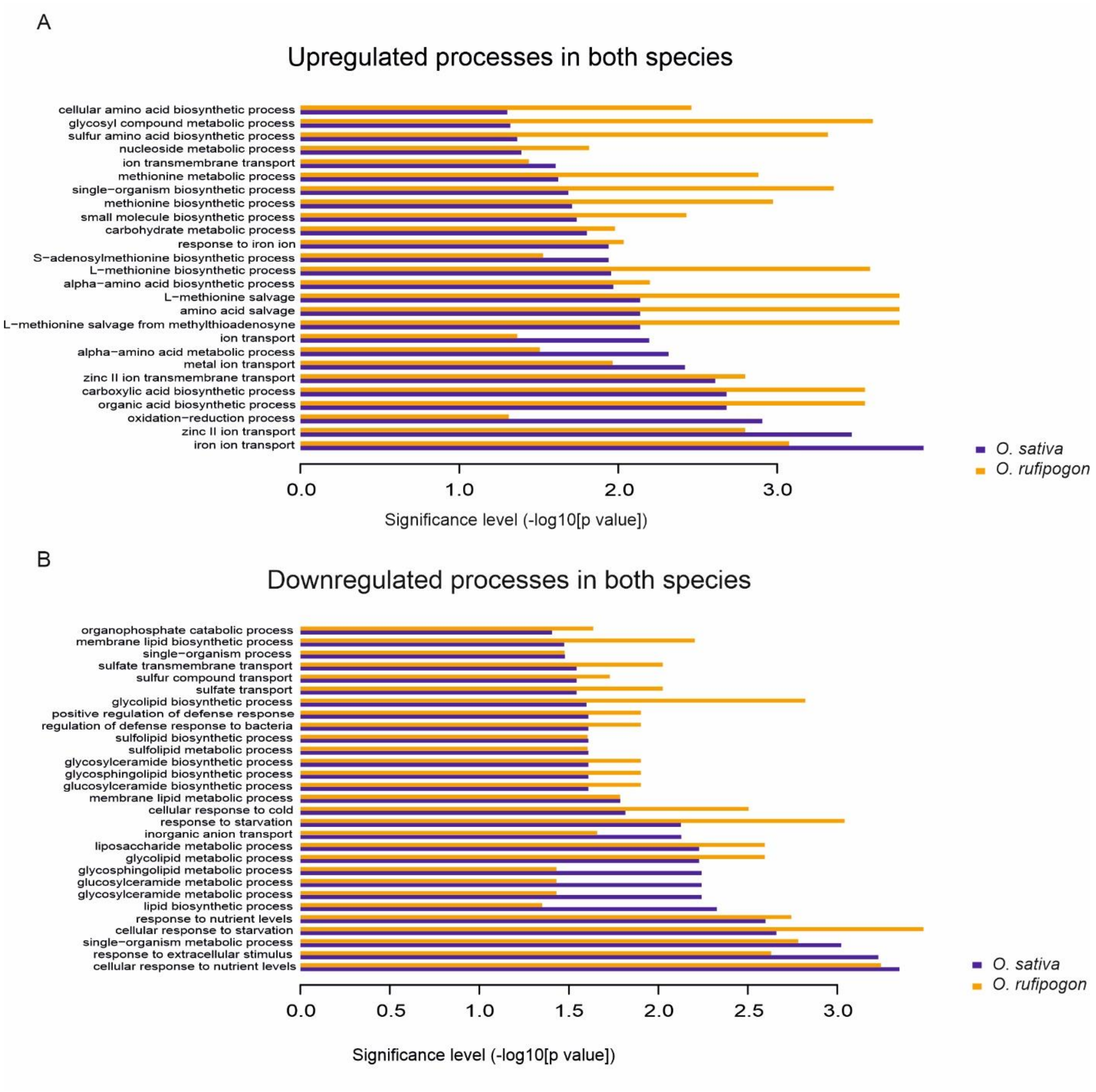

Supplementary Figure 3. Unique Gene Ontology (GO) terms in the response to Fe deficiency between cultivated (*Oryza sativa*) and wild (*Oryza rufipogon*) rice. Exclusive GO terms up and down-regulated by Fe deficiency in (A) *O. sativa* and (B) *O. rufipogon*. The figure presents the terms with high degree of significance for *O. sativa* and *O. rufipogon*. Blue bars represent terms up-regulated, and red bars represent terms down-regulated.

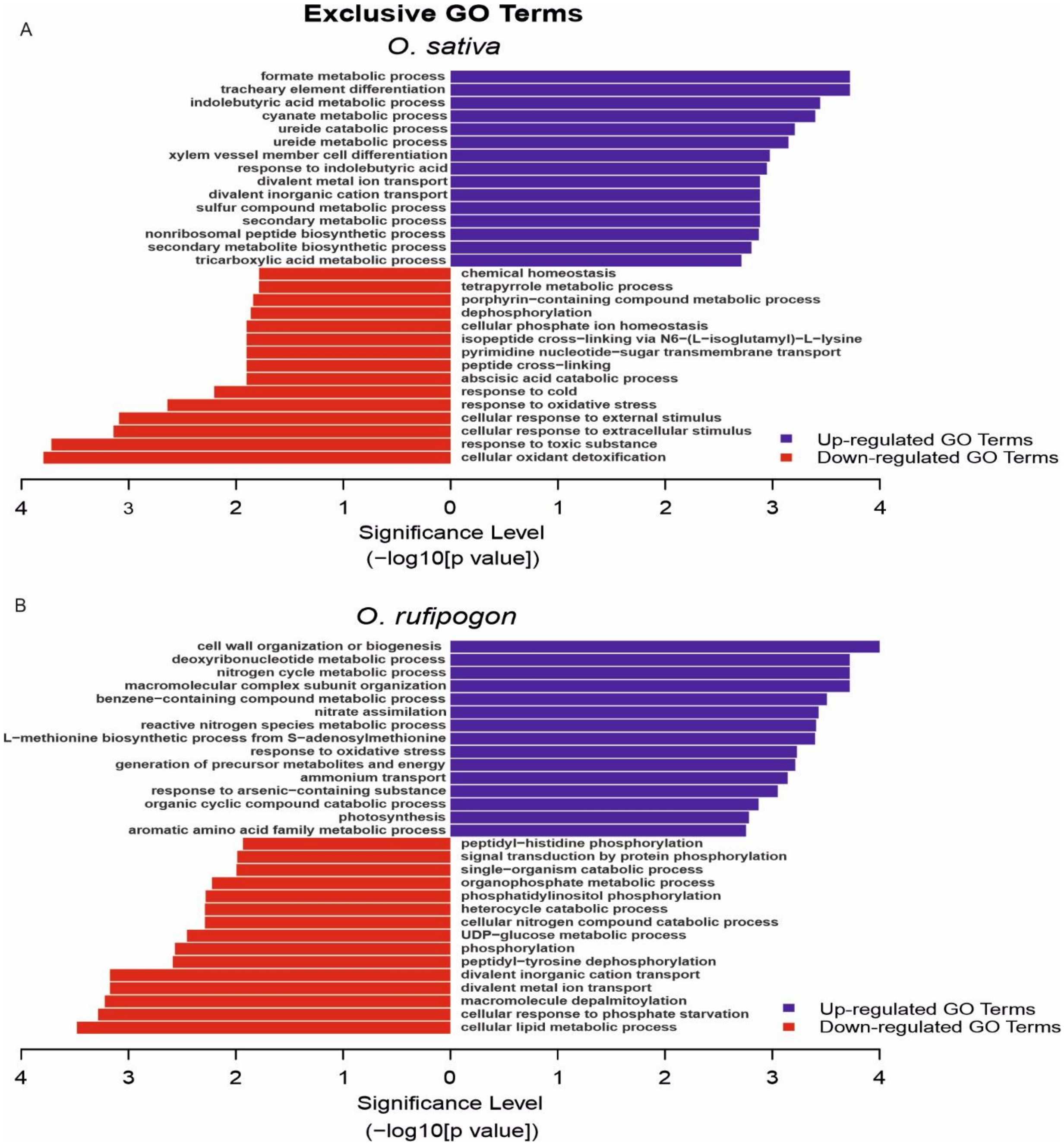

Supplementary Figure 4. Syntenic block search for the *OsIRT1* gene from *Oryza sativa*. A genomic block of *OsIRT1*, composed of 41 genes (20 genes up and 20 genes downstream of *OsIRT1*, plus *OsIRT1*), was employed to search the complete genomes of *Oryza meridionalis* (A) and *Oryza rufipogon* (B). In the *O. meridionalis* genome, no potential syntenic blocks were found (no peak occurs on the *y*-axis). When the *O. rufipogon* genome was considered as a positive control, potential syntenic blocks were found, indicated by a peak at the protein relative position 3000 (B).

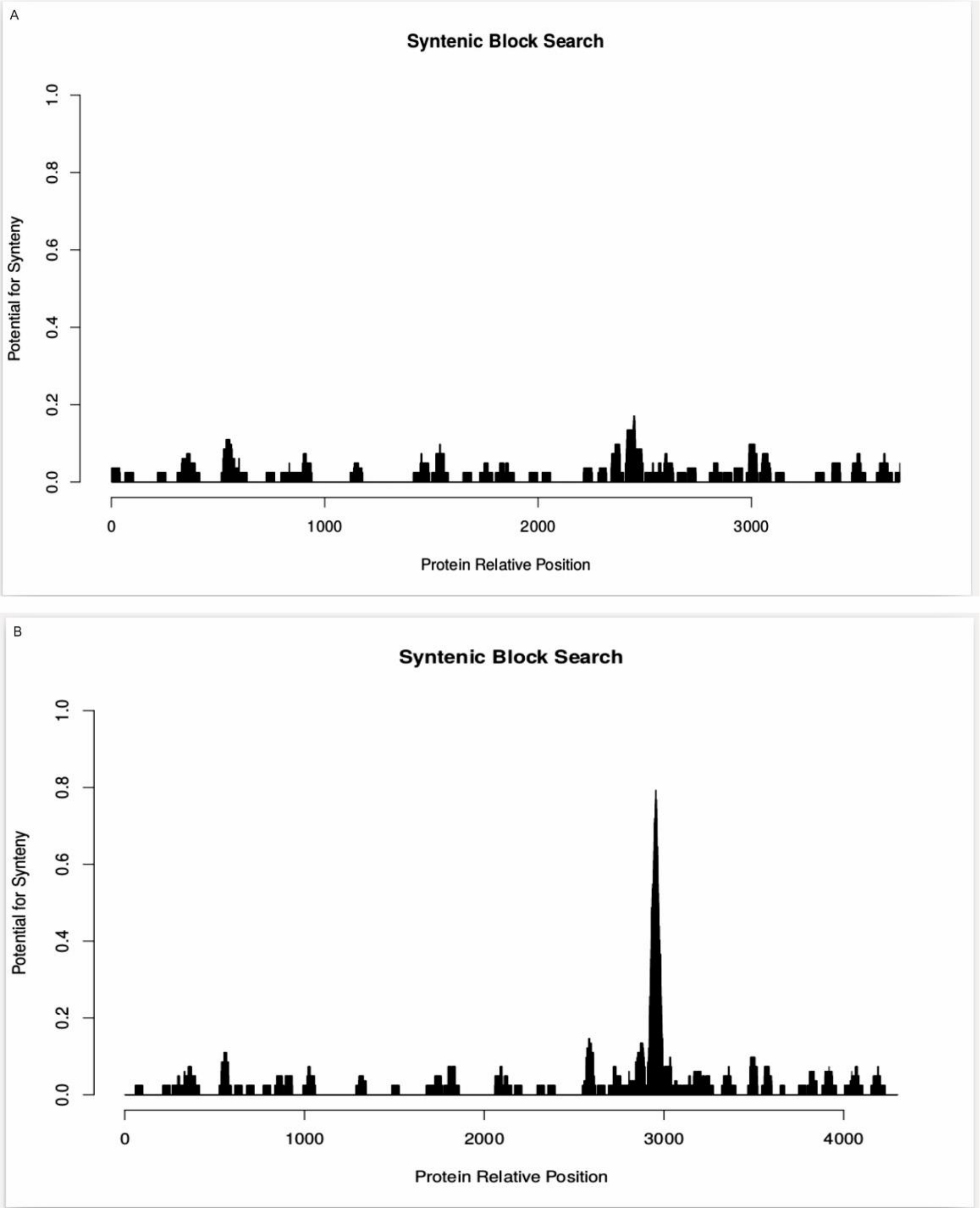

Supplementary Figure 5. Collinearity analyses of Fe deficiency-related genes in *Oryza spp.*, maize, and sorghum. Graphs displaying the results from the collinearity analysis, performed through the MCSCanX tool, across the *Oryza* genus, *Zea mays*, and *Sorghum bicolor* gene orthologs. The analyses were performed on genomic segments containing 41 genes. Arrows present the orientation of genes. Numbers represent the genes inside the genomic segments. The target gene (*YSL15*, *IRO2*, *NRAMP1*, or *IRT1*) is represented by number 0. (A) Collinearity analysis for *IRT1*. (B) Collinearity analysis for *YSL15*. (C) Collinearity analysis for *IRO2*. (D) Collinearity analysis for *NRAMP1*.

Genomic collinearity block

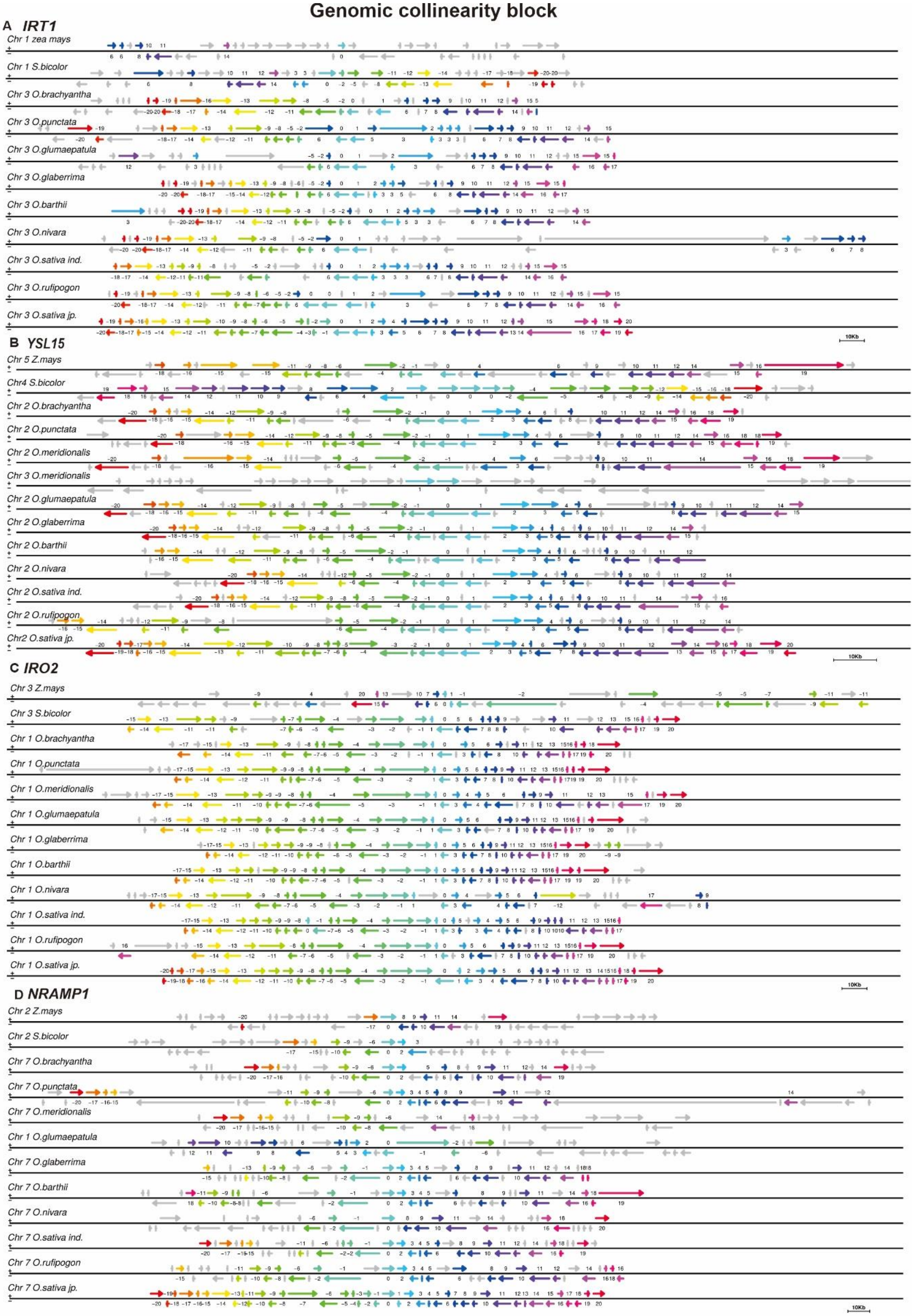

Supplementary Figure 6. Chlorophyll concentration in leaves of *Zea mays* plants grown under control (CC) or Fe deficiency (-Fe) conditions. Chlorophyll concentration (µg/mg DW). The *x*-axis represents days after the onset of -Fe treatment. Values are the averages of four samples ± SE (n = 4, 3 plants each). Asterisks indicate statistical difference between plants grown under CC and -Fe conditions (Student *t*-test, **P-value < 0.01). DW = dry weight.

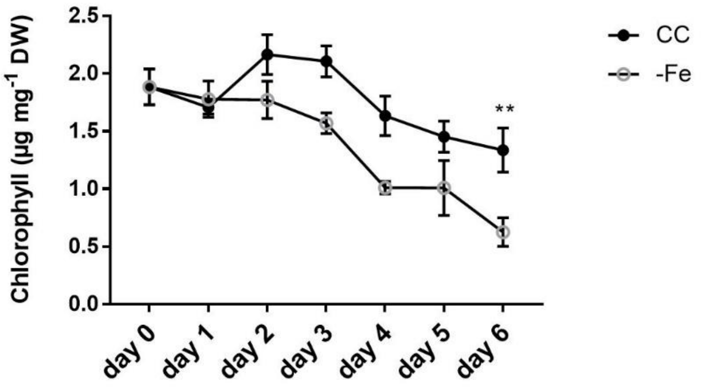

Supplementary Figure 7. Evaluation of Fe-deficiency symptoms when plants of *Sorghum bicolor* were grown under control (CC) or Fe deficiency (-Fe) conditions. (A) Chlorophyll concentration (µg/mg DW), (n = 4, 3 plants each). (B) Root dry weight (g) (n = 10). (B) Shoot dry weight (g) (n = 10). The *x*-axis represents days after the onset of -Fe treatment. Values are the averages of four or ten samples ± SE. Asterisks indicate statistical difference between plants grown under CC and -Fe conditions (Student *t*-test, *P-value < 0.05, **P-value < 0.01, ****P-value < 0.0001).

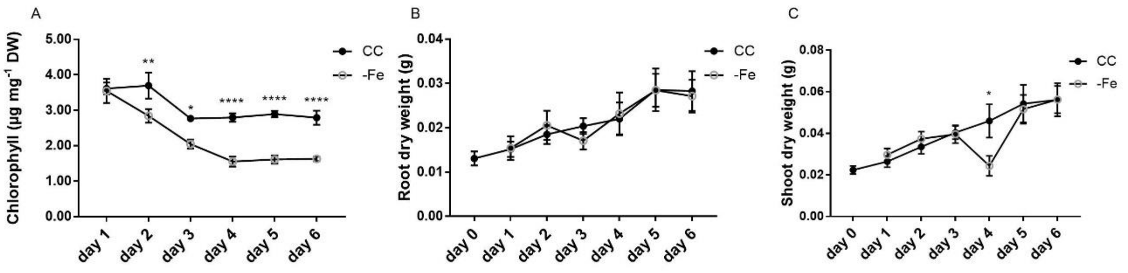

Supplement: Supplementary file 1 — Supplementary Material [file 41598_2019_52502_MOESM1_ESM.pdf]
